# Representing relations between individual contributions: when does joint action planning facilitate task performance?

**DOI:** 10.1007/s00426-026-02269-7

**Published:** 2026-03-03

**Authors:** Kassandra Friebe, Natalie Sebanz, Günther Knoblich

**Affiliations:** Social Mind and Body Group, Department of Cognitive Science, Central European University, Quellenstr. 51, Vienna, 1100 Austria

## Abstract

Understanding how individuals plan, predict and adapt their actions is central to understanding the high degree of coordination involved in joint action. While previous research has examined the role of joint action plans in linking individual action representations in a two-person joint action plan, the specific conditions and representations associated with these plans are less well understood. In this study, we investigated when and how joint action plans that specify the relations between individual contributions facilitate performance in a joint task. We hypothesized that joint action plans serve to organize individual contributions to a joint action by specifying the relations between them. Pairs of participants performed a joint computer task that required flipping blocks to produce a joint pattern. We manipulated the availability and information provided by two types of pre-cues: joint cues specified the relation between individual contributions and individual cues specified each participant’s individual part. The results of three experiments showed that information regarding the relation between individual contributions helped co-actors integrate representations of their individual contributions. This is evidenced by faster response times when a joint cue was presented together with a cue specifying the partner’s action. Moreover, joint cues could be used to compensate for the lack of information about individual contributions, as evidenced by faster response times when a joint cue was presented in trials without information about individual contributions. We conclude that joint action plans that specify the relations between individual contributions serve to organize representations of individual contributions and thereby help co-actors infer their own underspecified contribution to a joint action.

**Supplementary Information:**

The online version contains supplementary material available at 10.1007/s00426-026-02269-7.

## Introduction

On a daily basis, humans engage in coordinated actions with others to bring about joint outcomes (Sebanz et al., 2006; Sebanz & Knoblich, 2021). Such joint actions are crucial for many aspects of human social behaviour, including collaboration, teamwork, and communication. To function successfully, joint actions require coordination at multiple levels, as individual motor commands, action plans, perceptions, and intentions need to fit together (Knoblich et al., 2011). Many theories that aim to explain how joint coordination is achieved have focused on the level of intention and belief (Bratman, 1992; Gilbert, 1990; Pacherie, 2011) and the use of language in coordinating joint actions (Clark, 1996; Pickering & Garrod, 2004). Recent research has highlighted the processes that allow joint action partners to implement specific action plans (Butterfill & Sinigaglia, 2023; Vesper et al., 2010) and predict the effects of joint action (Pezzulo, 2013; Pezzulo et al., 2025; Sebanz & Knoblich, 2021). Theories of individual action planning claim that internal forward models in the motor system predict the outcomes of actions and compare them with real-world action effects (Wolpert & Flanagan, 2001; Wolpert & Ghahramani, 2000). Building on these theories, it has been proposed that joint action partners implement specific action plans to predict and adapt to a co-actor’s action (Friston & Frith, 2015; Pesquita et al., 2018; Sebanz & Knoblich, 2009; Wolpert et al., 2003). This hypothesis gained support from studies that demonstrate motor system involvement in planning and executing joint actions (Bolt & Loehr, 2024; Della Gatta et al., 2017; Hadley et al., 2015; Kokal et al., 2009; Newman-Norlund et al., 2008), including the accurate prediction of a co-actor’s movements during collaborative tasks (Vesper et al., 2014).

However, representing one’s own and a partner’s actions may be insufficient for the high degree of coordination required in joint actions. Previous research on joint action representations provides evidence that co-actors plan joint actions in a way that goes beyond individual planning. For example, in mimicry tasks, where co-actors show a heightened propensity to jointly mimic or synchronize with actions in response to movements performed by two individuals, as opposed to movements performed by a single individual (Ramenzoni et al., 2014; Tsai et al., 2011). Thus, even minimal models of joint action acknowledge the need for representations that go beyond the individual planning level (Butterfill, 2017; Vesper et al., 2010). A critical element is the concept of a joint action plan that specifies an outcome toward which individual actions are collectively oriented, as a single individual’s action directed toward that outcome would not suffice. Joint action plans have been shown to support coordination by linking individual actions into a “unitary two-person motor plan” (Sacheli et al., 2018), which enables individuals to predict and adapt to their partners’ contributions toward the joint outcome. While experimental evidence for the use of joint action plans has accumulated in recent years (Formica & Brass, 2024; Sacheli et al., 2018, 2021), it remains an open question whether co-actors generally prioritize joint planning, or primarily use joint-level information to optimize their own action.

On the one hand, co-actors may generally prioritize joint-level planning over individual-level planning, and thus always take into account information beyond the individual planning level.

On the other hand, co-actors may primarily optimize their own action, and use joint-level information when it helps them to plan their own action.

Evidence for the proposal that joint action planning is generally prioritized comes from studies showing that being instructed to act together rather than merely alongside one another can reduce interference (Clarke et al., 2019; Formica & Brass, 2024; Sacheli et al., 2018, 2021). Taking into account the joint level is thought to enhance the predictability of a partner’s action effects by linking them to the joint goal and to allow for the use of action-effect rules learned from one’s own individual motor experience. As a result, joint planning can help prevent interference caused by perceiving or anticipating a co-actor’s actions.

In support of this claim, Sacheli et al. instructed participants to jointly create a melody with a virtual partner by alternately performing congruent or incongruent actions (Sacheli et al., 2018). Notably, when participants were operating under a joint goal, the otherwise robust interference effects were eliminated. These results support the idea that joint action planning enables co-actors to integrate individual action representations with the representation of a joint goal, thus assuming a functional role in linking individual actions to a joint goal (Török et al., 2019).

There is also evidence that co-actors often incorporate joint-level information into their planning, even when it does not directly support (Kourtis et al., 2019) or may even interfere (Della Gatta et al., 2017) with the planning and execution of individual actions. Initial support for the prioritization of group-level representations under such conditions was provided by Kourtis et al. (2019), who employed a pre-cueing paradigm to investigate planning processes during joint actions. During pre-cueing tasks participants are asked to perform certain actions as quickly as possible. Just before instructions concerning the full action are given, some, all, or none of the action features may be specified in advance. The underlying logic is that the time required for performing a specific action reflects the time it takes to mentally represent the action parts that were not fully specified. This also implies that pre-cued information, which facilitates faster action initiation, is incorporated into the planning process. Previous research on pre-cueing individual actions has found that providing advance information about a particular action significantly reduces the reaction time required to perform it, as the person can prepare and initiate the motor response more efficiently (Rosenbaum, 1980; Rosenbaum & Kornblum, 1982). In Kourtis et al.’s study, participants performed synchronized actions to form different hand configurations together. Crucially, pre-cues that specified the relation between individual contributions (i.e., whether the same or different hand shapes were required) facilitated joint performance even in the presence of information about the individual action (e.g., hand facing forward or backward).

Together, these results show that co-actors tend to take into account joint-level information when engaging in joint actions, especially when it proves useful to planning and execution their own action or inferring something about a yet underspecified action at the group level (but see (Della Gatta et al., 2017).

It remains an open question whether joint action planning takes precedence over individual action by default or whether it is possible to ignore the joint planning level if focusing on individual contributions is more efficient. Specifically, in the study by Kourtis et al. (2019), the representation of the relation between individual contributions may have been beneficial due to the nature of the synchronization task, which required the coordination of body parts. Thus, it remains unknown whether co-actors generally process information about the relation between their own contributions and those of their partner or refrain from planning at the joint level when the task requires more distal effects and the focus is on individual reaction times rather than synchronization.

## The present study

The present study aimed to investigate when co-actors use joint and individual planning strategies to prepare for their own contribution to a joint action. We hypothesized that joint action planning can be used to organize relations between individual contributions to a joint action. This would allow co-actors to relate their own and others’ individual contributions to joint outcomes and to infer which individual contribution is required when a partner’s contribution is known or observed.

Specifically, we assume that while individual action planning allows co-actors to represent their own upcoming action, it does not allow them to represent how their own action and a partner’s action are related with respect to a joint outcome. Representing such relations therefore requires the joint planning level in which individual contributions are encoded as components of a joint action plan. For this reason, we use information about relations between individual contributions as an operationalization of joint-level representations.

Importantly, forming joint action plans may not always be necessary or efficient. When information about one’s own required action is fully specified, co-actors may rely primarily on individual action planning. In contrast, when information about a partner’s action and the relation between individual contributions can be integrated to determine one’s own contribution, joint action planning may provide a functional advantage. In this case, representations of the relation between individual contributions can be used to structure representations of both actions within a joint action plan, thereby supporting action selection and earlier motor preparation.

From this perspective, joint cues in our task allow us to test whether and when co-actors rely on joint-level action planning. If co-actors engage in joint action planning, relational cues should influence individual action preparation even when they do not directly specify the individual contribution.

**Fig. 1:**
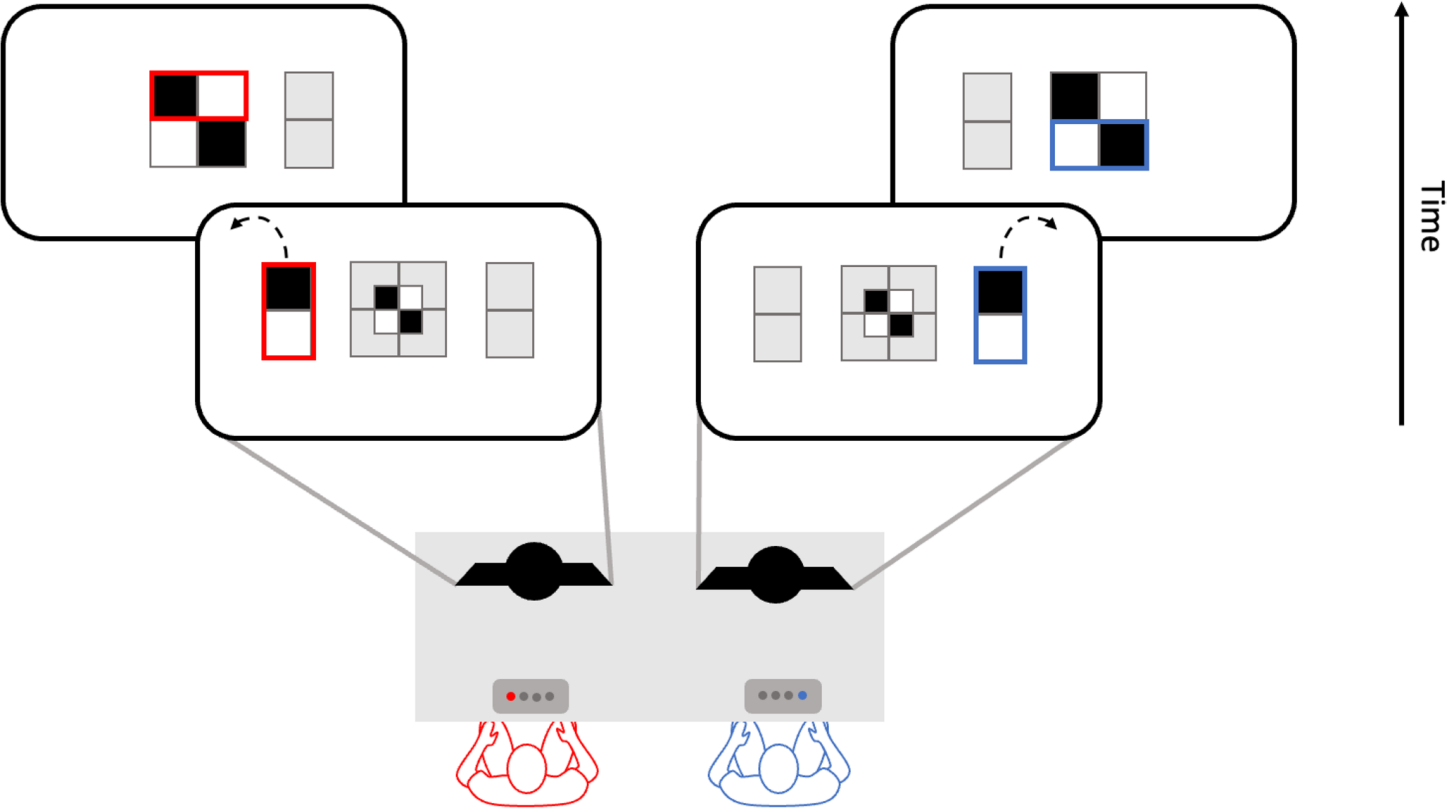
Schematic representation of the task. Participants performed the task jointly by rotating their individual block (presented on the far left side/far right side, see lower panels) either to the left or the right side to match the pre-defined pattern (shown in the middle, lower panels). The participant on the left side was responsible for flipping the rectangular block on the left to fill in the upper part of the grid (see upper panels). The participant on the right was responsible for flipping the rectangular block on the right side to fill in the lower part of the grid (upper panels). In the example illustrated here, the left participant needs to flip her block to the left while the right participant needs to flip her block to the right. Blocks were rotated to the left or right side by pressing the leftmost or rightmost button on a 4-button button box

We developed a new task that required two co-actors to rotate rectangular blocks to jointly create a square with a specific pattern of black and white elements (see Fig. 1). Before participants were informed about the pattern they were required to recreate, two consecutive pre-cues were presented to them: Joint cues informed them about the relation between individual contributions or remained non-informative, and individual cues informed them about their own or their partner’s contribution or remained non-informative.

*Experiment 1* investigated whether co-actors generally prioritize joint planning and take into account information about the relations between their individual contributions when engaged in a joint action. If so, a general benefit of receiving joint-level information should be observed. Alternatively, if co-actors primarily focus on optimizing their own actions, they may only consider joint-level information when it facilitates the preparation of their individual action. This could be achieved by using information on the relation between individual contributions to provide a structure into which representations of one’s own and a partner’s actions can be integrated, and from which one’s own contribution can be inferred. Accordingly, a benefit of receiving joint-level information should be observed when it enables individuals to infer their own contribution based on the representation of the partner’s contribution and the relation between them.

*Experiment 2* investigated whether joint action partners rely on joint action planning when there is uncertainty about the individual contributions required for a joint action. If information about individual contributions to a joint action becomes less reliable, participants may choose to ignore relational information and refrain from joint-level planning. Alternatively, information about the relation between individual contributions may be used to compensate for the increased uncertainty individual contributions, and thus increase the prevalence of joint action planning. To test these two alternative hypotheses, we varied the validity of individual cues in *Experiment 2*.

*Experiment 3* aimed to further investigate whether joint action planning requires relations between individual contributions be specified in advance of individual contributions to integrate information of a co-actor’s contribution. To this end, the order of joint and individual cues was randomized across blocks of trials.

## Experiment 1

Experiment 1 investigated whether co-actors represent their actions at the joint-level by default. If so, we should see that co-actors make use of information about the relation between individual contributions independently of the information provided by the individual cue. Under this account, we predicted a main effect of joint cue, reflected in faster reaction times and fewer errors when the relations between individual contributions (same or different actions required) are cued in advance, regardless of whether the individual cue specified one’s own action, the partner’s action, or remained non-informative.

Alternatively, if co-actors make use of joint-level information only to optimize their own action, we expect information about the relation between individual contributions to only benefit performance when it allows participants to infer something about their own underspecified action. Under this account, we predict an interaction between joint cue and individual cue, such that the effect of the joint cue would be absent or strongly reduced when the individual cue already specified one’s own action, but present when the individual cue specified the partner’s action.

### Method

#### *Transparency and openness*

We report how we determined our sample size, all data exclusions (if any), all manipulations, and all measures in the study. All data, preprocessing scripts, analysis code and research materials are available at: https://osf.io/7z3gn/?view_only=6bcf7d1f9da74a46be110bce62f68ce5. Data were analyzed using R, version 4.2.2. This experiment’s design and analysis plan were pre-registered at: https://aspredicted.org/18M_MCN. All deviations to the pre-registration are disclosed in the Methods section.

#### *Power analysis*

A power analysis was performed with G*Power 3 (Faul et al., 2007) to estimate the required sample size for all experiments reported in this manuscript. The analysis was conducted for a three-way repeated measures ANOVA with 12 within-subject measurements (2 × 2 × 3). We based our effect size estimate on previous experiments on joint action planning (Kourtis et al., 2014, 2019). Kourtis et al. (2019) reported a large effect size for the main effect of joint cue. However, as these experiments required participants to synchronize their movements and resulted in direct physical effects, whereas our task required only distal effects, the estimated effect size partial η² was 0.06, following Cohen’s (1988) convention for a medium effect, α was 0.05, β was 0.80, assumed correlation among repeated measures was 0, and non-sphericity correction ε was 1. This yielded a suggested sample size of 24 participants to achieve the desired power level. Reflecting on the pre-registered power analysis, we acknowledge that our choice to flatten the 2 × 2 × 3 design of our study to a 12-factor design and estimating the sample size based on a main effect likely resulted in a sample size too small to detect smaller interaction effects and increased the likelihood of a Type II Error.

A more appropriate workaround would have been to preserve the interaction structure by computing difference scores for the effect of joint cue presence separately for same and different action-relation conditions and deriving an interaction score from these contrasts. This interaction score could then be analyzed as the dependent variable in a one-way repeated-measures ANOVA across the three levels of the individual cue factor, thereby providing a closer approximation of the three-factor interaction within the constraints of G*Power. Using this alternative way of approximating the interaction structure, we recalculated the required sample size while keeping all other parameters constant. This analysis suggested a required sample size of 52 participants to detect medium-sized interaction effects (for G*power protocols and a mathematical justification, see Supplement E).

While we recognize the importance of studies with sufficient power, we have decided to proceed with the pre-registered sample size of 24 participants to ensure transparency and minimize the risk of bias arising from subsequent adjustments to the study design or sample size. A post hoc sensitivity analysis indicated that, given a sample size of 24, the study was powered to reliably detect effects of approximately f = 0.378 (corresponding to partial η² ≈ 0.125). Accordingly, smaller interaction effects may not have been reliably detectable, and non-significant interaction effects are therefore interpreted with appropriate caution.

#### *Participants*

Twenty-four participants (16 women) took part in the study. Participants were between 19 and 33 years old and the average age was 23.9 years (SD = 2.5). Pairs of participants were matched by handedness. The study was approved by the Ethical Research Committee of Central European University and performed in accordance with the Declaration of Helsinki. All participants gave their informed consent and were paid for their participation. This applies to all reported experiments. Data collection for all experiments took place in winter 2023/24.

Two participants exceeded the pre-registered exclusion criterion of committing more than 30% errors. However, our pre-registration did not explicitly specify whether such cases should result in the exclusion of individual participants or the entire pair. After careful consideration, we made a post-hoc decision to exclude the entire pair in such cases. Consequently, four participants were excluded and replaced in total. The pre-registrations for subsequent experiments were updated to explicitly state that the entire pair should be excluded under these circumstances.

#### *Apparatus and stimuli*

The experimental script was programmed and ran in Psychopy (v2022.2.5). Stimuli and instructions were displayed on two 21.5-inch monitors in black on a light gray background at a constant viewing distance of 70 cm. Participants responded using a four-button response box (manufactured by the Blackbox Toolkit).

#### *Procedure and stimuli*

Pairs of participants were seated side by side, each participant facing an individual computer screen (see Fig. 1). The participants were instructed to jointly recreate a pre-defined pattern made up of two rectangular black-and-white blocks that were arranged to form a larger black-and-white square. Each participant was responsible for rotating one of two blocks presented on the screen. The participant sitting on the left side of the table rotated the black-and-white block presented on the left side of the screen, that would fit the upper block of the larger square. Simultaneously, the participant sitting on the right side of the table rotated the black-and-white block on the right side of the screen, that would fit the lower block of the square. In order to recreate the pre-defined pattern, both participants needed to rotate their respective blocks by pressing the left or right key on their response box. Prior to seeing a Go-signal that fully specified the joint outcome to be achieved, i.e. that presented the black-and-white square participants had to recreate, two types of pre-cues were presented in sequential order, allowing participants to prepare some parts of the ensuing action earlier on:

A *joint cue* specified the relation between individual contributions or remained uninformative. On 50% of the trials, the joint cue specified the relation between individual contributions: The cue either indicted that the required contributions were the *same* – thus requiring the same action (both participants pressing the left key or the right key) and resulting in the same outcome (having two black and white blocks of the same orientation stacked upon each other) – or that they were different – thus requiring different actions (one participant pressing the left key while the other participant presses the right key) and resulting in different outcomes (having two black and white blocks of opposite orientation stacked upon each other). An equal sign (=) denoted that the required individual contributions from both participants were same. An unequal sign (≠) indicated that required individual contributions differed. On 50% of the trials the joint cue remained non-informative. A circle (○) acted as a cue to avoid perceptual effects in non-informative trials. The joint cue was presented at the center of the screen and obtained 2.4° × 2.4° of visual angle.

An *individual cue* specified one participant’s own task, the partner’s task or was non-informative. In 1/3 of the trials, the words “*your task*” were presented above a black and white rectangular block indicating the participant’s own required contribution, in 1/3 of the trials, the words “*your partner’s task*” were presented above a black and white rectangular block indicating the partner’s required contribution, and on 1/3 of trials no words were presented above a rectangular block made up of two gray square – the cue remained non-informative in these trails. The individual cue was presented at the center of the screen and obtained 4.9° × 2.4° visual angle.

**Fig. 2:**
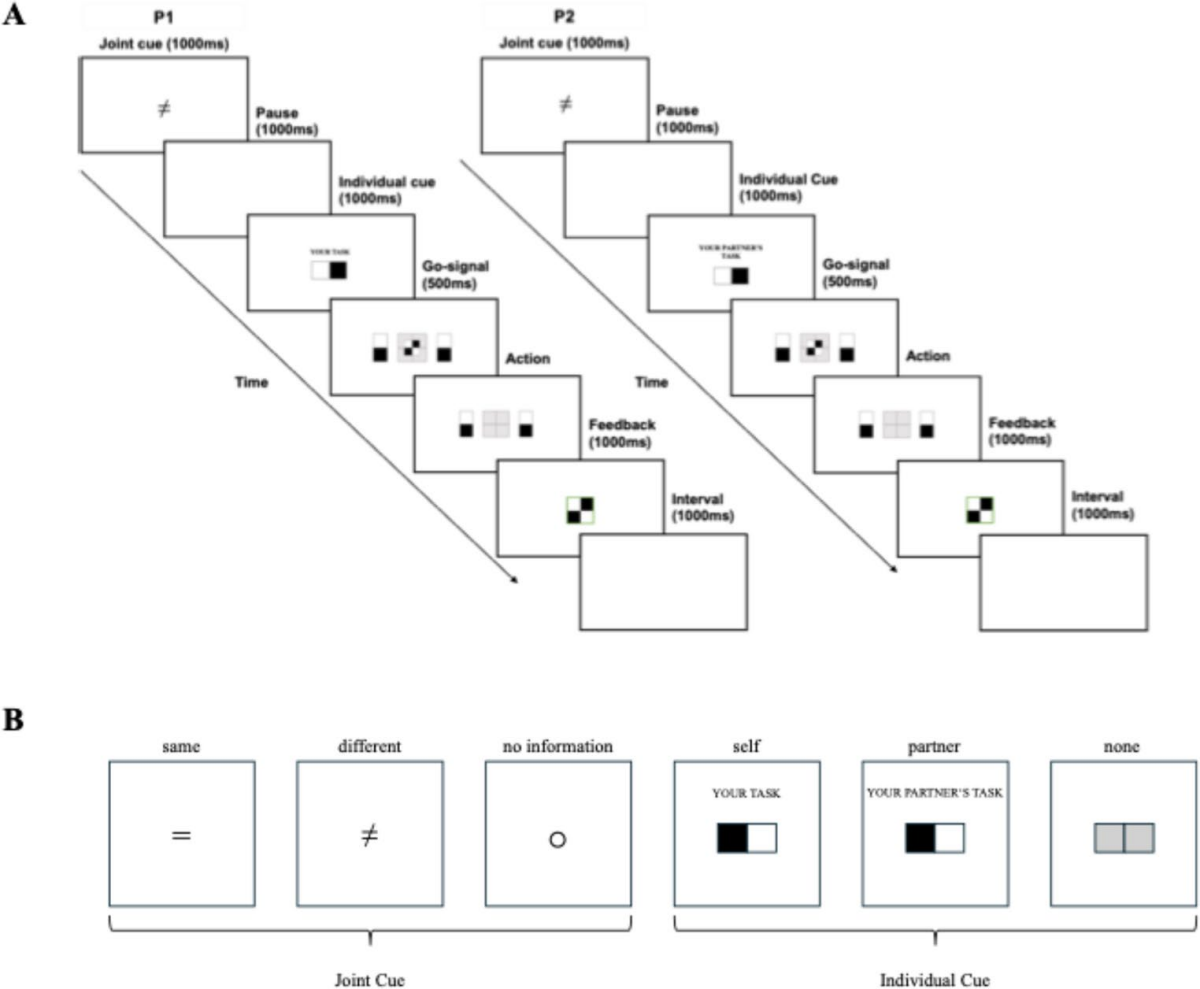
Experimental Trial Sequence and Types of Cues. **A** Trial sequence for Experiment (1) Each trial began with the presentation of a joint cue for 1000 ms, followed by a 1000 ms blank screen and the presentation of an individual cue for 1000 ms. Afterwards a Go-signal presented for 500 ms fully informed participants about the joint outcome to be achieved. Feedback was provided after action execution. **B** Different types of cues in Experiment 1 and (2) Joint cues informed participants about the relation of their individual contributions. An equal sign informed participants that the upcoming individual action contributions would be the same, an unequal sign informed them that the upcoming individual action contributions would be different, and a circle indicated that no advance information about the relation of individual action contributions was provided. Individual cues informed participants about their own contribution, their co-actor’s contribution or were non-informative

The order of cue presentation remained the same throughout the first experiment, with joint cues always preceding individual cues (see Fig. 2). Each pre-cue was presented for a duration of 1000 ms. There was a 1000 ms pause between cues, during which the screen remained blank. After the two pre-cues, a Go-signal informed the participants of the pattern they were to reproduce. A small square consisting of four smaller squares was presented for 500 ms in the center (4.9° × 4.9°) above a larger square (9.8° × 9.8°) and colored in black and white to inform participants of the pattern they had to recreate by rotating their individual blocks, which were presented at a visual angle of 28.8° × 9.8° from the center. After the presentation of the Go-signal, the participants initiated their actions. Following participants’ actions feedback was provided. Feedback was given for both correct and incorrect trials. A green or red frame around the square indicated whether both co-actors had reacted correctly or incorrectly. A red frame was followed by individual feedback, which indicated who of the two co-actors had rotated the block incorrectly, by means of another red or green frame around the individual block.

There were 4 blocks of 96 trials (384 trials total) separated by short pauses. Within each block 8 trials of each experimental condition appeared in random order. The experimental conditions resulted from the combination of the three factors *joint cue* (present vs. absent), *individual cue* (self vs. partner vs. non-informative) and *action-relation* (same vs. different).

Before performing the joint task, participants completed a short practice block that matched the procedure of experimental blocks,,so that the participants could familiarise themselves with the sequence of the task.

### Results

We calculated repeated measures analysis of variance (ANOVA; Greenhouse–Geisser corrected). Post-hoc tests were conducted using Bonferroni-corrected paired t-tests.

Prior to statistical analysis, we excluded all trials for each participant in which responses were 2.5 standard deviations faster or slower than the mean RT of each participant and condition, resulting in an exclusion of 3.4% of trials. For each participant, individual mean response times (RT) and error rates (ER) for each experimental condition were calculated (see Supplement A).

Although our pre-registration indicated that exclusion would be based on the individual mean of participants, in the actual analysis exclusions were determined based on the mean of participant conditions to reflect our overall analysis plan. To appropriately weight and combine error rates and response times, we calculated Inverse Efficiency Scores (Bruyer & Brysbaert, 2011; IES), using the formula RT/(1-ER). By weighing RT with ER, IES provides a measure that neutralizes potential speed-accuracy trade-offs and allows for an interpretation that takes into account RT and ER at the same time. When comparing conditions with the same RT but different ER, the IES is lower for conditions with smaller ER and higher for conditions with larger ER. Thus, a lower IES corresponds to overall better task performance, while a higher IES corresponds to poorer task performance. As a Shapiro Wilk normality test indicated non-normality in the data set (see Table C.1. in Supplement C), a log transformation was applied to the data set prior to statistical analysis. After log transformation, Shapiro–Wilk tests indicated that all condition-wise distributions met normality assumptions (all ps > 0.05; see Table C.1 in Supplement C). After log transformation, Shapiro–Wilk tests indicated that all condition-wise distributions met normality assumptions (all ps > 0.05; see Table C.1 in Supplement C).

**Fig. 3:**
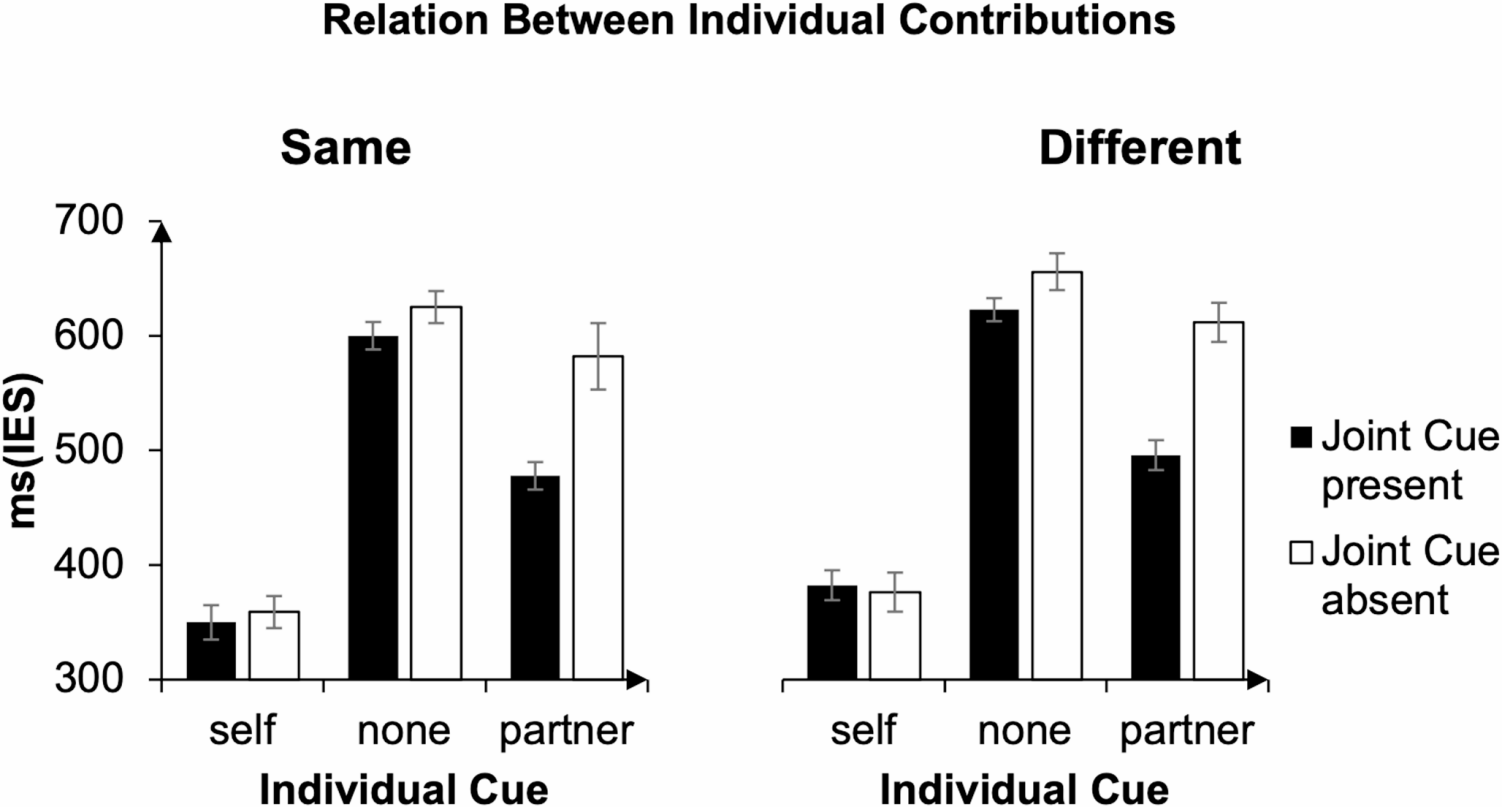
Mean Inverse Efficiency Scores (IES) for the ‘same action’ and the ‘different action’ condition for Experiment 1. When the individual cue provided information about the partner’s action or remained non-informative, participants benefited from information about the action-relation provided by the joint cue. Error bars represent standard errors of the means, with the Cosineau–Morey correction applied (Morey, 2008)

*Inverse Efficiency Scores (IES).* Figure 3 displays inverse efficiency scores as a function of individual cue and joint cue separately for joint cues that required the same action (Panel A left) and joint cues that required a different action (Panel B, right). Note that the joint cue was present in 50% of the trials (black bars in both panels) and absent in 50% of the trials (white bars in both panels).

The pre-registered three-way repeated-measures ANOVA with the factors joint cue (present vs. absent), action-relation (same vs. different), and individual cue (self vs. partner vs. non-informative) revealed a significant main effect of joint cue, F(1, 23) = 21.66, *p* < .001, partial η² = 0.485 (joint cue present: M = 488 ms, SD = 172 ms; absent: M = 534 ms, SD = 199 ms). A main effect of individual cue (F(1.39, 31.88) = 149.12, *p* < .001, partial η² = 0.866)) indicated that IES was smaller in self trials (M = 367 ms, SD = 125 ms), than in partner trials (M = 541 ms, SD = 179 ms, t(95) = 15.4, *p* < .001, Cohen’s *d* = 1.57), and smaller in partner trials compared in non-informative trials (M = 625 ms, SD = 153 ms, t(95) = 9.00, *p* < .001, Cohen’s *d* = 0.919). A significant main effect of action-relation (F(1, 23) = 18.43, *p* < .001, partial η² = 0.445) indicates that task performance was better when the required contributions were same (M = 499 ms, SD = 188 ms), than when they were different (M = 523 ms, SD = 187 ms).

The ANOVA also revealed a significant joint cue × individual cue interaction, F(1.60, 36.87) = 22.96, *p* < .001, η² = 0.500, indicating that the effect of joint cue was modulated by individual cue. Follow-up comparisons indicate that the presence of the joint cue benefitted task performance in the partner trials, t(47) = 7.17, *p* < .001, Cohen’s *d* = 1.04, with smaller IES when a joint cue was present (M = 486 ms, SD = 155 ms), than when it was not (M = 596 ms, SD = 186 ms), and in the non-informative individual cue condition, t(47) = 2.94, *p* = .001, Cohen’s *d* = 0.42, with smaller IES when the joint cue was present (M = 611 ms, SD = 144 ms), than when it was not (M = 640 ms, SD = 162 ms). The presence of a joint cue did not significantly benefit task performance in self trials, t(47) = 0.11, *p* = .911, Cohen’s *d* = 0.02.

To directly test the advantage of joint cues, we computed for each participant an index reflecting the effect of joint cues by subtracting performance in the absence of a joint cue from performance in the presence of a joint cue^1^. One-sample t-tests against zero showed that joint cues significantly improved performance both when the individual cue was non-informative, t(23) = − 2.33, *p* = .029, d = 0.48, and when it specified the partner’s contribution, t(23) = − 5.68, *p* < .001, d = 1.16. Critically, a paired-samples t-test comparing the joint-cue advantage between the two conditions revealed that the effect of joint cues was significantly larger in the partner-cue condition than in the non-informative condition, t(23) = − 5.04, *p* < .001, 95% CI [− 0.24, − 0.10], d ≈ 1.05.

All other two-way interactions and the three-way interaction were not significant, all ps > 0.05, see Table B.1. in Supplement B.

Lastly, to assess whether the observed interaction patterns were driven disproportionately by response speed or accuracy, we additionally analyzed response times and error rates separately. The critical joint cue × individual cue interaction was replicated in RTs, mirroring the reported IES pattern, whereas error rates showed a similar trend, but no significant interaction (see Supplement B, Tabel B.2 and B.3). This pattern indicates that the IES effects primarily reflect differences in response preparation rather than speed–accuracy trade-offs.

### Discussion

Experiment 1 investigated the role of joint action plans that specify relations between individual contributions. We hypothesized that representing these relations would make it easier to infer one’s own task from the partner’s task. Our findings corroborate this hypothesis, showing that participants make use of joint cues to infer their own contribution to a joint action from a partner’s contribution.

Crucially, although to a lesser extent, participants also benefited from relational information when the individual cue was non-informative. That is, even when the joint cue did not allow participants to infer anything about their own contribution by integrating joint- and individual-level information (as is possible with partner cues), knowing the relation between individual contributions still facilitated performance. This pattern suggests that when uncertainty about one’s own contribution cannot be reduced directly, co-actors may instead use relational information to reduce uncertainty at the joint level by narrowing down the set of action alternatives that need to be prepared for (Kourtis et al., 2019).

Consistent with a large number of previous studies (Jentzsch et al., 2004; Leuthold & Jentzsch, 2009; Rosenbaum, 1980; Rosenbaum & Kornblum, 1982; Scheibe et al., 2009), we also found that advance information about an individual action to be performed accelerates action initiation. Advance information about the relation between one’s own and a partner’s contribution to a joint action neither interfered with this benefit nor did it provide an additional benefit. Thus, it seems that individuals ignore or cannot benefit from relational information when their own contribution is directly specified.

## Experiment 2

Experiment 1 demonstrated that joint action partners use relational information in joint action plans to infer their own contribution or guide action planning in absence of information of individual contributions. Experiment 2 investigated whether relational information about individual contributions is still coded in joint action plans when there is uncertainty about the individual contributions required for a joint action. If information about individual contributions to a joint action, particularly the partner’s, becomes less reliable, participants may choose to ignore relational information and not form joint action plans. This account predicts no main effect of joint cue and no interaction between joint cue and individual cue in the 75% validity block, while replicating the effects observed in Experiment 1 in the 100% validity block.

Alternatively, relational information about individual contributions may be used to compensate for the increased uncertainty about one’s own and a partner’s individual contribution. Under this account, joint cues should produce a main effect or interact with individual cue in the 75% validity block, indicating that relational information continues to influence action preparation despite reduced reliability of individual cues. Accordingly, this account predicts a three-way interaction between block validity, joint cue, and individual cue, reflecting differential use of relational information as a function of cue reliability.

To test these two alternative hypotheses, we varied the validity of individual cues in Experiment 2. In one condition, cues to one’s own and to the partner’s contributions were 100% valid. For this condition we expected to replicate the results of Experiment 1. In the other condition, cues to one’s own and to the partner’s contribution were only valid in 75% of the trials. If relational information about individual contributions is used to compensate uncertainty about each individual contribution, knowing in advance whether one’s own and a partner’s individual contributions are the same or different should improve performance. Alternatively, if relational information is discarded, implying that no joint action plans are formed, joint cues that specify the relation between individual contributions should not affect performance.

### Method

The methods of Experiment 2 were the same as in Experiment 1, with the following exceptions:

- *Transparency and openness.* Design and analysis plan for Experiment 2 were pre-registered at: https://aspredicted.org/QWQ_ZPD.
- *Power analysis.* In our previous experiment, a sample size of 24 participants, organized into 12 pairs, provided robust statistical power for detecting the main effect of joint cue presence as well as substantial effect sizes associated with other relevant factors. We maintained the same sample size for Experiment 2, as this likely allows for replication of the previously observed effects, at least in the 100% individual cue validity condition.
- *Participants.* Twenty-four right-handed subjects (20 women) with an average age of 25.4 years (SD = 3.3), ranging from 18 to 30 years. No dyad met the exclusion criterion of one individual having an over-all error rate of > 30%. However, one dyad had to be excluded and replaced nonetheless, as one participant had already taken part in an earlier version of the experiment.
- *Procedure.* The four experimental blocks of Experiment 2 were divided into two larger blocks based on cue validity: One set with 75% individual cue validity and another with 100% individual cue validity. This organization ensured that participants engaged in two blocks where individual cues were completely reliable (100% validity) and two blocks where cues were reliable 75% of the time. Thus, there were 2 experimental blocks of 96 trials (192 trials in each set, 384 trials in total) with 75% individual cue validity and 100% individual cue validity each. Within each block 8 trials of each experimental condition appeared in random order. The experimental conditions resulted from the combination of the three factors *joint cue* (present vs. absent), *individual cue* (self vs. partner vs. non-informative) and *action-relation* (same vs. different).

Participants were always informed about the type of block they were engaging in, and the order of these blocks was counterbalanced across participants. Joint cues were always reliable. A training threshold at 70% accuracy was introduced to account for the relatively high error rates in Experiment 1 and a trial timeout after 3 s was introduced to speed up participants’ responses.

### Results

#### *Cue validity effect*

A paired sample t-test was conducted for the 75% individual cue validity block as a manipulation check to determine if invalidly cued trials resulted in higher error rates. The results indicated a statistically significant difference in error rates by cue validity, t(23) = 2.82, *p* = .01. Specifically, error rates were larger when the cue was invalid (11.2%), than when it was valid (2.8%).

#### *Inverse Efficiency Score*

Prior to statistical analysis, we excluded all trials for each participant that were incorrectly cued, as well as correctly cued trials in which responses were 2.5 standard deviations faster or slower than the mean RT of each participant and condition (3.5%). In a next step, the data was split into the data from blocks of 100% and 75% individual cue validity respectively. As a Shapiro Wilk normality test indicated non-normality for both data sets (see Table C.2. in Supplement C), a log transformation was applied to the data set prior to statistical analysis.

Departing from the pre-registered analyses, we conducted a three-way ANOVA between the factors individual cue × joint cue × block validity to more closely resemble our study design.

The main effect of joint cue was significant, F(1, 23) = 9.15, *p* = .006, partial η² = 0.285 (joint cue present: M = 554 ms, SD = 171 ms; absent: M = 575 ms, SD = 181 ms). Moreover, a main effect of individual cue, F(1.3, 29.9) = 30.26, *p* < .001, partial η² = 0.568, indicated that the type of individual cue had a significant influence on IES with smaller IES when participants’ own contribution was specified (M = 492 ms, SD = 136 ms), than when their partner’s contributions was specified (M = 578 ms, SD = 184 ms, t(95) = 7.26, *p* < .001, Cohen’s *d* = 0.74), and smaller IES when their partner’s contribution was specified, than when no information about individual contributions was provided (M = 623 ms, SD = 180 ms, t(95) = 6.25, *p* < .001, Cohen’s *d* = 0.67).

The individual cue × block validity interaction was significant, F(1.24, 28.60) = 6.22, *p* = .01, partial η² = 0.213.

The three-way interaction of individual cue × joint cue × block validity was significant F(1.74, 39.98) = 3.91, *p* = .03, partial η² = 0.145.

The other interactions and the main effect of block validity were not significant, see Supplement B.

To make the three-way interaction easier to interpret, we performed follow-up two-way ANOVAs separately for the two block validity conditions, in line with our pre-registered analyses. To this end, the data was split into the data from blocks of 100% and 75% individual cue validity respectively. As a Shapiro-Wilk normality test indicated non-normality of both datasets (see Table C.2. in Supplement C), a log transformation was applied to the data set prior to statistical analysis. After log transformation, Shapiro–Wilk tests indicated that all condition-wise distributions met normality assumptions (all ps > 0.05; see Table C.2 in Supplement C).

**Fig. 4:**
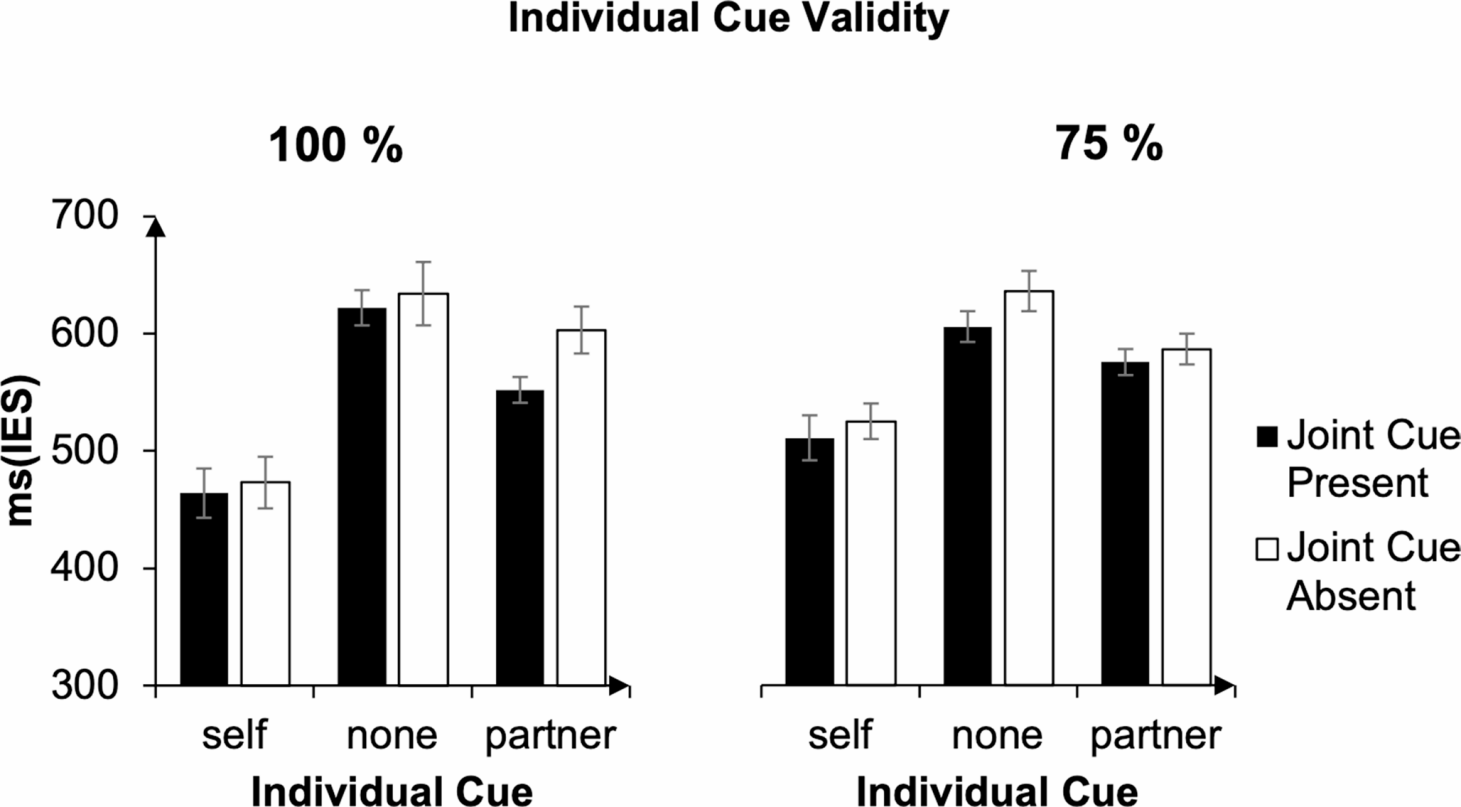
IES for the ‘100% individual cue validity block’ and the ‘75% individual cue validity block’ for Experiment 2. Participants benefited from information about the relation between individual contributions provided by the joint cue. This effect was especially strong when the individual cue provided information about the partner’s action in the 100% individual cue validity block. Error bars represent standard errors of the means, with the Cosineau–Morey correction applied (Morey, 2008)

Similar to the pattern of results of Experiment 1, participants’ IES in the 100% individual cue validity condition (left in Fig. 4) were smaller when their own and their partner’s action was specified than when no individual contribution was cued, as indicated by a main effect of individual cue, F(1.26, 28.97) = 27.18, *p* < .001, partial η² = 0.542, with smaller IES in self trials (M = 469 ms, SD = 155 ms), compared to partner trials (M = 577 ms, SD = 212 ms, t(47) = 6.21, *p* < .001, Cohen’s *d* = 0.90), and smaller IES in partner trials than in non-informative trials (M = 0.628, SD = 0.203, t(47) = 4.94, *p* < .001, Cohen’s *d* = 0.71).

IES were smaller when information about the relation between individual actions was cued (M = 546 ms, SD = 196 ms) than when it wasn’t (M = 570 ms, SD = 207 ms), as indicated by a main effect of joint cue, F(1, 23) = 6.53, *p* = .018, partial η² = 0.221.

The joint cue × individual cue interaction was significant, F(1.91, 43.97) = 4.91, *p* = .013, partial η² = 0.176. Follow-up comparisons indicated that the presence of a joint cue especially benefitted performance when the partner’s contribution was specified, t(23) = 3.75, *p* = .001, Cohen’s *d* = 0.77, as indicated by smaller IES when it was present (M = 552 ms, SD = 196 ms)), than when it was not (M = 603 ms, SD = 228 ms). All other effects were not significant (all ps > 0.05).

The right panel of Fig. 4 displays the IES of the 75% individual cue validity block as a function of individual cue and joint cue. IES were smaller when information about the relation between individual actions was provided (M = 562 ms, SD = 143 ms) by the joint cue than when it wasn’t (M = 580 ms, SD = 152 ms), as indicated by a main effect of joint cue, F(1, 23) = 4.39, *p* = .047, partial η² = 0.16.

Participants’ IES also varied with differing individual cues, as indicated by a main effect of individual cue, F(1.4, 32.1) = 15.96, *p* < .001, partial η² = 0.41. IES were smaller when participants’ own contribution was cued (M = 516 ms, SD = 11 ms), than when their partner’s contribution was cued (M = 578 ms, SD = 155 ms, t(47) = 4.19, *p* < .001, Cohen’s *d* = 0.6), and smaller when their partner’s contribution was cued than when no information about individual contributions was specified (M = 618 ms, SD = 155 ms, t(47) = 4.23, *p* < .001, Cohen’s *d* = 0.61). Unlike for the blocks with 100% valid individual cues, the two-way interaction between the factors joint cue and individual cue was not significant, *p* > .05.

Bayesian model comparison yielded positive evidence against the individual cue × joint cue interaction, as the additive model (BF = 9.23 × 10⁷) was approximately seven times more likely than the interaction model (BF = 1.31 × 10⁷), indicating that the interaction term did not improve model evidence beyond the main effects.

Separate analyses of reaction times and error rates showed that the three-way interaction between joint cue, individual cue, and block validity observed in IES was reproduced in reaction times (see Supplement B, Table B.5), but not in error rates, which were overall low across conditions. (see Supplement B, B.6).

### Discussion

In Experiment 2 we investigated how information about relations between individual contributions to a joint action is used in joint action planning when information about individual contributions is not fully reliable.

The results of Experiment 2 suggest that joint action partners also consider information about relations between individual contributions under uncertainty about individual contributions. However, our results show that the facilitative role of joint action plans in organizing partner contributions and thereby reducing uncertainty about one’s own contribution to a joint outcome was especially strong when joint action partners could rely on the information about individual contributions being always reliable.

Besides replicating the advantage of receiving information about the relation between individual contributions in partner trials in the 100% individual cue validity condition, Experiment 2 demonstrated that information about the relation between individual contributions facilitated participants’ action planning by itself, even when information about individual contributions was not always reliable. This finding indicates that joint action partners represent joint actions at a level that goes beyond the representation of individual contributions alone. When acting jointly with a partner, action planning seems to be driven by joint action plans that represent relations between individual contributions, thereby allowing one to prepare for a limited amount of possible motor responses, resulting in faster and more accurate responses.

Similar to Experiment 1, faster responses after a joint cue in the 100% individual cue validity condition were mainly driven by faster responses in the partner condition compared to the self and non-informative condition. These results point to the role of joint action plans in organizing representations of individual contributions that allow co-actors to reliably infer information about their own tasks.

In contrast to Experiment 1, the results in the 75% individual cue validity condition did not show as strong a tendency towards joint cues improving action planning when a partner’s action was specified, compared to one’s own or no information. Instead, there was a general tendency towards representing relations between individual contributions that benefitted response times.

## Experiment 3

This experiment aimed to further investigate how flexibly individual contributions can be integrated into joint action plans. One possibility is that joint action plans need to be specified in advance of the individual contributions to adopt an organizing function and help integrate representations of a co-actor’s contribution. Alternatively, information about individual contributions and their relations may be flexibly integrated. In this case the order in which information is presented should not influence task performance.

Accordingly, if joint action plans need to be specified in advance to take on an organizing function, RTs should be faster and error rates should be lower if relations are cued before individual contributions. This account predicts a three-way interaction between the factors joint cue, individual cue, and cue order. Follow-up analyses of this interaction should show that the joint cue × individual cue interaction is present only when the joint cue is presented first (replicating the pattern observed in Experiment 1). If co-actors can flexibly integrate individual contributions and their relations, a joint cue should lead to faster RTs and fewer errors even if information about individual contributions is provided in advance. Under this account, we predicted a joint cue × individual cue interaction that does not depend on cue order.

### Method

The methods in Experiment 3 were the same as in Experiment 2, with the following exceptions:

- *Transparency and openness.* The design and analysis plan for Experiment 3 were pre-registered at: https://aspredicted.org/VXS_Q1F.
- *Power analysis.* In Experiment 1 and 2, a sample size of 24 participants provided robust statistical power for detecting the main effect of joint cue presence and relevant interactions. We decided to maintain the same sample size for Experiment 3.
- *Participants.* Twenty-four right-handed subjects (21 women) with an average age of 24.3 years (SD = 2.5), ranging from 21 to 33 years. One dyad met the exclusion criterion of at least one co-actor having an error rate > 30%. This dyad was excluded and replaced.
- *Apparatus and stimuli.* In contrast to the previous experiments, only two types of individual cues were presented in Experiment 3. Individual cues either specified the partner’s contribution or remained non-informative. In partner trials, both participants saw the required contributions of their partner, whereas in the non-informative trials neither of them was informed about individual contributions. Experiments 1 and 2 showed that joint action plans that specify relations between individual contributions mainly help to integrate partner contributions but are not utilized if one’s own contribution is clearly defined. Our primary interest was therefore to understand how the representation of relations between individual contributions might facilitate the organization of representations of a partner’s contribution. Due to this design change, both participants always saw the partner cues at the same time, such that partner trials created the possibility that participants could infer their own required contribution from the cue presented on their partner’s screen. To ensure that inferences about one’s own contribution relied solely on the experimentally manipulated cues, an occluder was placed between the two screens. This was not necessary in Experiments 1 and 2, because whenever one participant received a partner cue, the other participant simultaneously received a self cue, such that viewing the partner’s screen did not provide additional information.
- *Procedure.* Differing from the first two experiments in this series, the order in which pre-cues were presented was counterbalanced across experimental blocks. This allowed us to investigate whether relational information about individual action contributions needs to be specified prior to information about specific individual contributions or whether relational information can be effective even when the partner’s action has already been specified. Experiment 3 consisted of 4 experimental blocks of 96 trials (384 trials in total). Within each block, the order of trials was randomized, but cue order was held constant within a block. The experimental conditions resulted from the combination of the three factors *joint cue* (present vs. absent), *individual cue* (partner vs. non-informative) and *cue order* (joint cue first vs. joint cue second).

### Results

#### *Inverse Efficiency Scores*

Prior to statistical analysis, we excluded all trials in which responses times were 2.5 standard deviations faster or slower than the mean RT of each participant and condition (3.1%). As a Shapiro Wilk normality test indicated non-normality for the data set (see Table C.3. in Supplement C), a log transformation was applied prior to statistical analysis. After log transformation, Shapiro–Wilk tests indicated that all condition-wise distributions met normality assumptions (all ps > 0.05; see Table C.3 in Supplement C).

**Fig. 5:**
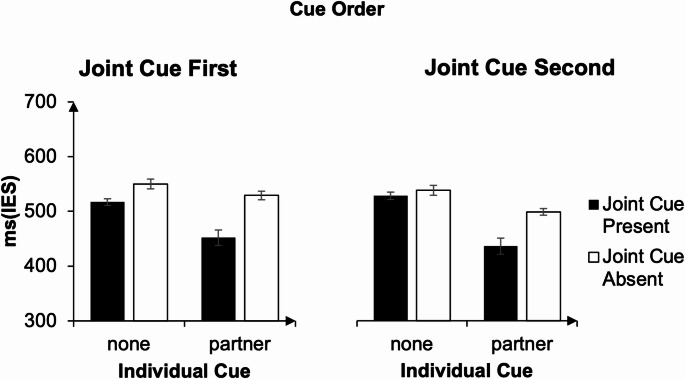
IES for the ‘joint cue first order’ and the ‘joint cue second order’ in Experiments 3. IES were significantly smaller when a joint cue was presented in combination with an individual cue. Error bars represent standard errors of the means, with the Cosineau–Morey correction applied (Morey, 2008)

Figure 5 displays IES as a function of individual cue and joint cue, separated by cue order.

Results of the pre-registered 3-way ANOVA between the factors joint cue (present vs. absent), individual cue (partner vs. non-informative), and cue order (joint cue first vs. joint cue second) revealed a significant main effect of joint cue, F(1, 23) = 16.97, *p* < .001, partial η² = 0.425 (joint cue was present: M = 482 ms, SD = 155 ms, joint cue absent: M = 528 m, SD = 124 ms).

There was also a significant main effect of individual cue, F(1, 23) = 24, *p* < .001, partial η² = 0.511, showing that IES were smaller when the partner’s task was cued (M = 478 ms, SD = 150 ms) than when it was not (M = 532 ms, SD = 128 ms).

A significant main effect of cue order, F(1, 23) = 9.36, *p* = .006, partial η² = 0.289, indicated that participants’ IES were smaller when the joint cue was presented second (M = 499 ms, SD = 141 ms), compared to when it was presented first (M = 0.512, SD = 0.143).

The two-way interactions between joint cue × individual cue (F(1, 23) = 13.17, *p* = .001, partial η² = 0.364), joint cue presence × cue order (F(1, 23) = 4.9, *p* = .004, partial η² = 0.176), and individual cue × cue order (F(1, 23) = 9.99, *p* = .004, partial η² = 0.303) were all significant.

Follow-up tests indicate that the advantage of the joint cue is significant for both types of individual cue, with faster response times in the partner condition when a joint cue is present (M = 444 ms, SD = 169 ms) than when it is absent (M = 513 ms, SD = 119 ms, t(47) = 5.55, *p* < .001, Cohen’s *d* = 0.8), and faster response times in the non-informative condition when a joint cue is present (M = 521 ms, SD = 13 ms) than absent (M = 543 ms, SD = 119 ms, t(47) = 3.48, *p* = .001, Cohen’s *d* = 0.5).

Following up the joint cue × cue order interaction shows that presenting the non-informative joint cue prior to the individual cue resulted in larger IES (M = 540 ms, SD = 121 ms), than presenting the joint cue after the individual cue (M = 516 ms, SD = 127 ms, t(47) = 4.02, *p* < .001, Cohen’s *d* = 0.58). This effect was not significant for the informative joint cue (M*diff* = 0.003, t(47) = 0.47, *p* = .64, Cohen’s *d* = 0.07).

Lastly, following up the individual cue × cue order interaction reveals that IES were significantly smaller when a partner cue was presented prior to a joint cue (M = 466 ms, SD = 149 ms) than when it was presented later (M = 491 ms, SD = 152 ms, t(47) = 4.35, *p* < .001, Cohen’s *d* = 0.63). This difference was not significant for non-informative individual cues (M*diff* = 2 ms, t(47) = 0.14, *p* = .89, Cohen’s *d* = 0.02).

The three-way interaction between the factors joint cue, individual cue and cue order was not significant, F(1, 23) = 0.51, *p* = .48, partial η² = 0.022. A complementary Bayesian model comparison further indicated that the data were better explained by models including only lower-order terms (BF = 3.31 × 10¹⁵) than by a model additionally including the three-way interaction (BF = 9.20 × 10¹³), with the lower-order model being approximately 36 times more likely. Thus, while the three-way interaction cannot be ruled out in principle, its inclusion did not improve model evidence beyond the lower-order effects.

As an additional check on the contribution of speed and accuracy to the IES results, we examined reaction times and error rates separately. The IES pattern of significant main effects and two-way interactions, as well as the absence of a three-way interaction, was mirrored in the reaction time analysis (see Supplement B, Table B.14). Error rates did not show a comparable pattern of main effects or two-way interactions. However, the three-way interaction was significant, F(1, 23) = 4.45, *p* = .03, partial η² = 0.191 (see Supplement B, Table B.15).

### Discussion

The results of Experiment 3 show that co-actors flexibly integrated representations of individual contributions to a joint action plan, so that joint action plans can be used to help specify aspects of participants’ own unspecified action independently of the order information was presented in. Consistent with Experiment 1 and 2, participants’ IES were significantly smaller when information about the relation between individual contributions was provided together with information about the partner’s contribution or a non-informative individual cue, than when it was not. Importantly, the results of Experiment 3 showed that this advantage was independent of the order in which cues were presented. Our findings indicate that joint action plans retain their organizing function independently of whether information about the relation of individual contributions is specified before or after information about individual contributions.

Finally, in trials where information about the relation between individual contributions was absent, we found that participants’ IES were smaller when information about their co-actor’s task was presented earlier than later. This finding is in line with previous research on action planning indicating that earlier information about features of a task result in faster response times as participants can prepare for the ensuing task from earlier on (Jentzsch et al., 2004; Leuthold & Jentzsch, 2009; Rosenbaum, 1980; Rosenbaum & Kornblum, 1982; Scheibe et al., 2009).

While our sample size was pre-registered based on the sample size considerations for Experiment 1 and 2, we acknowledge that the changes in the design of Experiment 3 introduced additional factors and modified the relevant factor levels. As a result, the original power analysis does not fully match with these modifications. Specifically, the introduction of the new factor *cue order* and removal of one level of the factor individual cue, were not sufficiently considered when pre-registering our study. A possible consequence of this oversight is an increased risk of Type II Errors, which means that some minor interaction effects may not have been detected. However, it is important to note that the primary interactions central to our hypotheses, as well as all main effects, were detected with sufficient effect sizes. Although we did not detect a three-way interaction, the direction of the joint cue × individual cue interaction is the same for both cue conditions. Thus, while we acknowledge this limitation, our central findings remain interpretable and theoretically meaningful.

## General discussion

The present study set out to investigate whether co-actors generally prioritize joint action planning, or whether they flexibly switch between joint and individual planning levels depending on what best supports their own performance. To this end, we designed a pre-cueing experiment in which certain aspects of the upcoming joint action were cued prior to a fully specifying Go-signal. Joint cues informed participants about the relation between individual contributions, while individual cues informed them about either their own or their partner’s contribution to the joint goal or remained non-informative. If co-actors generally prioritize joint action planning over individual action planning, joint cues should provide a performance benefit independently of the information provided by the individual cue. Alternatively, if co-actors switch between joint and individual planning to optimize their own performance, joint cues should be especially helpful when they allow co-actors to infer something about their own contribution.

Across three experiments, we consistently found evidence supporting the hypothesis that co-actors take into account joint-level information when it helps them optimize their own action, rather than showing a general prioritization of joint over individual action planning. That is, co-actors used joint-level information when it provided a benefit for planning and executing their individual contribution to a joint goal, but did not appear to prioritize joint action planning by default. Specifically, information about the relation between individual contributions was useful when it helped participants infer something about their own underspecified action.

In *Experiment 1*, participants only benefited from joint-level cues when the individual cue was either non-informative or indicated the partner’s required action. When participants received a cue specifying their own action, joint-level information provided no additional benefit. This suggests that relational information was disregarded when it was redundant for preparing one’s own contribution. Crucially, the effect of joint-level cues did not depend on whether individual contributions were the same or different, indicating that the facilitative role of joint planning is not tied to a specific type of action relation, but rather to its role in reducing uncertainty about one’s own contribution.

Similarly, in *Experiment 3*, we found that the effectiveness of joint cues did not depend on cue order. Participants benefited from relational information regardless of whether it was presented before or after the individual cues. This indicates that joint- and individual-level information can be flexibly integrated during the planning process. Again, joint cues provided a performance benefit when paired with information about the partner’s contribution or with a non-informative individual cue.

*Experiment 2* extended these findings in a different direction by examining how the validity of individual-level information influences co-actors’ use of joint-level planning. When information about individual contributions became less reliable (75% valid), participants were less likely to selectively use information about the relation between individual contributions to infer their own required action from information about their partner’s contribution. This may reflect that while co-actors represent information about individual contributions and the relations between them, they invest fewer cognitive resources in integrating these cues to make inferences about their own action when the available information is unreliable.

One crucial finding concerns trials in which the joint cue was paired with a non-informative individual cue. Even in these cases, participants showed performance benefits from the joint cue, despite the fact that it did not allow them to infer anything about their own contribution by integrating joint- and individual-level information (as is possible with partner cues). Knowing only the relation between individual contributions still enables co-actors to reduce the number of action alternatives they must prepare for at the group level (Kourtis et al., 2019). The present finding suggests that when uncertainty about one’s own contribution cannot be reduced, co-actors may instead shift to reducing uncertainty at the joint level. The fact that participants’ own actions were not affected by the presence of a joint cue when their individual contribution was specified by the individual cue suggests that the co-actors flexibly refrained from joint action planning when task demands did not require them to do so, because they were able to plan their own contribution without taking the joint planning level into account. Previous work has shown that joint action planning modulates how a partner’s actions are represented, such that integrating the partner’s contribution within a joint action plan can reduce interference and improve action efficiency (Clarke et al., 2019; Formica & Brass, 2024; Sacheli et al., 2018, 2021). The present results extend this work by showing how information about the relation between one’s own and a partner’s contribution can be used to guide the planning of one’s own action. Specifically, knowledge about the relation between one’s own and the partner’s actions modulated performance depending on whether it supported inference about one’s own required contribution (partner cue trials) or instead primarily reduced uncertainty at the joint level (non-informative individual cue cues). At the same time, when one’s own contribution was explicitly specified, co-actors did not benefit from additional information about the relation between individual contributions, suggesting that joint-level information is used selectively when it provides added value for planning one’s own action. It is important to consider the role played by the specific cues used in our experiment. Recent arguments suggest that it is not the particular type of information conveyed by the cues, but rather their role in priming early preparation for joint action planning, that accounts for the advantages provided by joint cues. According to the literature on priming in joint action (Molden, 2014), this perspective holds that any cue presented early in the process could prime the preparation of a joint action, thereby improving performance regardless of its specific content. From this view, joint cues should generally prime co-actors to engage in joint action planning and enhance performance independent of their informational content. In contrast to this assumption, our findings support the interpretation that co-actors actively utilize the content of the joint cue. First, a main effect of action relation across all three experiments indicates that the cue’s content is indeed processed. Second, at the time the joint cue is presented, it is still uncertain whether the subsequent individual cue will be self-related, partner-related, or non-informative. If the effect of the joint cue were purely due to early action priming, we would expect a similar performance advantage in self trials. However, this was not the case. The absence of a main effect of joint cues in self trials suggests that their impact extends beyond mere facilitation of early planning. Further evidence for this interpretation comes from the finding that the joint cue remains effective even when presented after an informative individual cue. If its function were limited to early priming, it would become redundant once the individual cue had already initiated the planning process. Yet, as demonstrated in *Experiment 3*, the joint cue still provides a significant additional advantage, even when it follows the individual cue. This suggests that the information conveyed by the joint cue plays a crucial role in enhancing joint action performance. We therefore propose that while early priming of joint action planning, as observed in *Experiment 3* when informative cues (joint cue or partner cue) are provided early, supports earlier preparation at the joint level (Kourtis et al., 2019), the content of the cue is also processed and may specifically help to integrate other types of information later in the planning process. In the case of the joint cue, knowing the relationship between individual contributions appears to refine the representation of each actor’s role, ultimately leading to better performance.

The present findings raise several important questions for future research. One key issue concerns the nature and scope of joint action planning. While our study focused on how co-actors use information about the relation between individual contributions, it remains an open question which other aspects of a joint action are also represented. A joint cue in our experiment informed participants that their individual actions, and their outcomes were the same or different. Previous research has shown that joint action plans are tied to shared goals (Sacheli et al., 2018), but that co-actors also represent the actions and errors of their partners and adapt accordingly (Formica & Brass, 2024; Sacheli et al., 2021). For example, in domains like ensemble music performance, where co-actors must coordinate not only their discrete actions but also expressive features such as timing, dynamics, and phrasing, it is likely that more aspects need to be represented at the group-level.

Furthermore, the importance of joint-level planning may vary depending on the spatiotemporal constraints of the joint action. In tasks that require tight temporal and spatial coordination, co-actors may prioritize joint planning earlier or more strongly than in loosely coordinated tasks. Indeed, this may help explain differences across studies: while our task allowed for temporal flexibility in planning, other paradigms (Kourtis et al., 2019; Sacheli et al., 2018) may require early prioritization of joint plans due to stringent coordination demands. Accordingly, the present findings should be generalized with caution to tightly synchronized joint actions, as joint action planning might take precedence here to afford tight coordination (see for example Kourtis et al., 2019).

Lastly, it remains unclear whether a more general underlying mechanism accounts for the observed effects. One possible interpretation is that our findings do not reflect a social effect, but rather stem from the fact that both participants were informed about the relation between their required contributions and the individual cue presented. From this perspective, the joint cue could be understood as a task-reversal cue that allows participants to infer their own contribution from the information provided by the individual cue without considering their partner’s action. As such, our main finding would still hold and indicate that co-actors take into account information beyond the individual planning level to optimize their own action, as in the case where they use relational information to infer their own required contribution from the representation of a partner’s contribution. However, this task-reversal explanation cannot account for one key aspect of our results: as previously argued (Kourtis et al., 2019), the task reversal interpretation of the joint cue cannot account for its observed advantage in conditions where individual contributions are not specified (see *Experiments 1* and *Experiment 3*). No task can be reversed here that would allow participants to infer information about their own required contribution. Nonetheless, further research is necessary to explore and clarify the nature of this mechanism.

Although partners received different cues and were not required to respond simultaneously, the task nevertheless involved a dyadic interaction structure, as individual actions jointly determined a shared outcome. However as both participants were instructed to respond as quickly as possible individually, we assumed that the response times of the two participants in a pair are independent.

Although partners received different cues and were not required to respond simultaneously, the task nevertheless involved a dyadic interaction structure, as individual actions jointly determined a shared outcome. In the present design, partners did not receive identical cues on a given trial, which made a fully dyadic trial-level analysis infeasible. At the same time, participants’ responses were likely not fully interdepended within pairs. Such coupling implies reduced variance in our statistical analyses. Importantly, however, our primary inferences concern within-participant effects of experimentally manipulated cue information. These effects are derived from within-person contrasts across conditions and are therefore not explained by dyad-level similarities in baseline performance.

Finally, we acknowledge that limitations in statistical power constrain the interpretation of some of our findings. The original power analysis was based on a simplified approximation of the factorial design and was used to determine the sample size for all three experiments. In Experiments 2 and 3, this approach was retained despite changes in design structure and in the theoretically relevant interaction effects. Following best practice, effect sizes observed in Experiment 1 should ideally have been used to inform new a priori power analyses for the subsequent experiments. Post-hoc sensitivity analyses suggest that smaller interaction effects may not have been reliably detectable. Consequently, null results, especially for higher-order interactions, should be interpreted with caution and not as definitive evidence for the absence of underlying effects. Our Bayesian analyses for the non-significant two-way interaction in the 75% individual cue validity condition in Experiment 2 and the three-way interaction in Experiment 3 provided evidence in favour of models excluding these interaction terms (i.e., evidence against including the interactions), rather than merely reflecting a lack of sensitivity to detect them. Our Bayesian analyses for the non-significant two-way interaction in the 75% individual cue validity condition in Experiment 2 and the three-way interaction in Experiment 3 suggest that the data provide evidence against the presence of these interaction effects (i.e., in favour of models without the interaction terms), rather than simply being underpowered to detect them.

In conclusion, our results suggest that co-actors can flexibly switch between joint and individual action planning depending on task demands. In our dyadic computer task, which did not require close spatiotemporal coordination, joint action planning proved particularly beneficial when it enabled co-actors to infer their own required contribution based on the specified action of their partner or reduce uncertainty at the group-level. This benefit appears to arise when joint action planning specifies the relation between individual contributions in a way that allows each co-actor to deduce their own role from the other’s assigned action or reduce the number of action alternatives the group needs to prepare for.

## Supplementary Information

Below is the link to the electronic supplementary material.

Supplementary Material 1

## Data Availability

All data, preprocessing scripts, analysis code and research materials are available at: https://osf.io/7z3gn/?view_only=6bcf7d1f9da74a46be110bce62f68ce5.

## Footnotes

1 We thank an anonymous reviewer for this suggestion.
