# Supplementary material for: Representing relations between individual contributions: when does joint action planning facilitate task performance?
Source: Psychol Res. 2026 Mar 3;90(2):51. doi: 10.1007/s00426-026-02269-7 (PMC12957024; doi:10.1007/s00426-026-02269-7)
Supplement: Supplementary file 1 — Supplementary Material 1 [file 426_2026_2269_MOESM1_ESM.docx]

**Supplementary Material**

**Supplementary Material A**

Table A.1. Descriptive Statistics Experiment 1

| Condition | Accuracy | RT (ms) | IES (ms) |
| --- | --- | --- | --- |
| JP – Non-Inf – Diff | 0.957 ± 0.203 | 590 ± 243 | 621 ± 140 |
| JP – Non-Inf – Same | 0.857 ± 0.203 | 570 ± 233 | 600 ± 149 |
| JP – Partner – Diff | 0.930 ± 0.257 | 456 ± 258 | 495 ± 159 |
| JP – Partner – Same | 0.949 ± 0.220 | 450 ± 232 | 478 ± 154 |
| JP – Self– Diff | 0.964 ± 0.188 | 368 ± 221 | 382 ± 124 |
| JP – Self – Same | 0.983 ± 0.129 | 343 ± 206 | 350 ± 122 |
| JA – Non-Inf – Diff | 0.944 ± 0.230 | 606 ± 242 | 654 ± 158 |
| JA – Non-Inf – Same | 0.957 ± 0.203 | 587 ± 235 | 625 ± 168 |
| JA – Partner – Diff | 0.910 ± 0.286 | 540 ± 248 | 610 ± 185 |
| JA – Partner – Same | 0.909 ± 0.288 | 509 ± 185 | 582 ± 190 |
| JA – Self– Diff | 0.987 ± 0.113 | 369 ± 237 | 376 ± 135 |
| JA – Self – Same | 0.977 ± 0.151 | 349 ± 204 | 359 ± 123 |

*Note.* Group means and standard deviations of all dependent measures for each cell of the 3 (Individual Cue: Self vs. Partner vs. Non-Informative) x 2 (Joint Cue: Joint Cue Present vs. Joint Cue Absent) x 2 (Action/Relation: Same vs. Different) experimental design. RT = response time; IES = inverse efficiency scores; JP: Joint Cue Present; JA: Joint Cue Absent; Non-Inf: Non-Informative Individual Cue

Table A.2. Descriptive Statistics Experiment 2

| Condition | Accuracy | RT (ms) | IES (ms) | |
| --- | --- | --- | --- | --- |
| *100 % Individual Cue Validity* | | | |  |
| JP – Non-Inf | 0.971 ± 0.167 | 596 ± 240 | 622 ± 213 | |
| JP – Partner | 0.954 ± 0.209 | 516 ± 209 | 552 ± 196 | |
| JP – Self | 0.967 ± 0.178 | 448 ± 206 | 464 ± 149 | |
| JA – Non-Inf | 0.974 ± 0.159 | 616 ± 244 | 634 ± 196 | |
| JA – Partner | 0.943 ± 0.233 | 555 ± 214 | 603 ± 228 | |
| JA – Self | 0.980 ± 0.138 | 463 ± 222 | 473 ± 164 | |
| *75 % Individual Cue Validity* | | | | |
| JP – Non-Inf | 0.974 ± 0.159 | 584 ± 208 | 603 ± 148 | |
| JP – Partner | 0.977 ± 0.149 | 556 ± 221 | 572 ± 157 | |
| JP – Self | 0.974 ± 0.159 | 496 ± 168 | 509 ± 105 | |
| JA – Non-Inf | 0.971 ± 0.167 | 611 ± 231 | 633 ± 163 | |
| JA – Partner | 0.960 ± 0.196 | 554 ± 178 | 584 ± 156 | |
| JA – Self | 0.972 ± 0.164 | 507 ± 175 | 523 ± 117 | |

*Note.* Group means and standard deviations of all dependent measures for each cell of the 2 (Individual Cue Validity Block: 100 % Individual Cue Validity vs. 75 % Individual Cue Validity) x 2 (Joint Cue: Joint Cue Present vs. Joint Cue Absent) x 2 (Action/Relation: Same vs. Different) experimental design. RT = response time; IES = inverse efficiency scores; JP: Joint Cue Present; JA: Joint Cue Absent; Non-Inf: Non-Informative Individual Cue

Table A.3. Descriptive Statistics Experiment 3

| Condition | Accuracy | RT (ms) | IES (ms) | |
| --- | --- | --- | --- | --- |
| *Joint Cue First* | | | |  |
| JP – Non-Inf | 0.974 ± 0.159 | 501 ± 174 | 517 ± 138 | |
| JP – Partner | 0.974 ± 0.159 | 438 ± 217 | 452 ± 174 | |
| JA – Non-Inf | 0.972 ± 0.164 | 534 ± 178 | 550 ± 126 | |
| JA – Partner | 0.985 ± 0.121 | 521 ± 161 | 529 ± 117 | |
| *Joint Cue Second* | | | | |
| JP – Non-Inf | 0.968 ± 0.176 | 508 ± 174 | 526 ± 126 | |
| JP – Partner | 0.982 ± 0.134 | 427 ± 209 | 435 ± 168 | |
| JA – Non-Inf | 0.977 ± 0.151 | 523 ± 174 | 536 ± 130 | |
| JA – Partner | 0.975 ± 0.157 | 485 ± 161 | 497 ± 122 | |

*Note.* JP: Joint Cue Present; JA: Joint Cue Absent; Non-Inf: Non-Informative Individual Cue; Diff: Different

**Supplementary Material B**

Table B.1. ANOVA Table Experiment 1, main analysis IES

| Effect | *df1* | *df2* | *F* | *p* | *Partial η²* |
| --- | --- | --- | --- | --- | --- |
| Joint Cue | 1.00 | 23.00 | 21.663 | <.001 | 0.485 |
| Individual Cue | 1.39 | 31.88 | 149.119 | <.001 | 0.866 |
| Action-Relation | 1.00 | 23.00 | 18.433 | <.001 | 0.445 |
| Joint Cue × Individual Cue | 1.60 | 36.87 | 22.957 | <.001 | 0.500 |
| Joint Cue × Action-Relation | 1.00 | 23.00 | 0.225 | .64 | 0.010 |
| Individual Cue × Action-Relation | 1.70 | 39.04 | 0.411 | .632 | 0.018 |
| Joint Cue × Individual Cue × Action-Relation | 1.96 | 45.02 | 1.105 | .339 | 0.046 |

*Note.* The two-way interaction of joint cue x individual cue, and the main effects of joint cue, individual cue and action-relation were significant. All other interactions were not significant. Greenhouse-Geisser corrections were applied.

Table B.2. ANOVA Table Experiment 1, RT

| Effect | *df1* | *df2* | *F* | *p* | *Partial η²* |
| --- | --- | --- | --- | --- | --- |
| Joint Cue | 1.00 | 23.00 | 20.551 | <.001 | 0.472 |
| Individual Cue | 1.30 | 29.92 | 263.055 | <.001 | 0.920 |
| Action-Relation | 1.00 | 23.00 | 10.428 | .004 | 0.312 |
| Joint Cue × Individual Cue | 1.76 | 40.37 | 15.905 | <.001 | 0.409 |
| Joint Cue × Action-Relation | 1.00 | 23.00 | 0.648 | .429 | 0.027 |
| Individual Cue × Action-Relation | 1.64 | 37.77 | 0.206 | .206 | 0.009 |
| Joint Cue × Individual Cue × Action-Relation | 1.99 | 45.71 | 1.618 | .210 | 0.066 |

*Note.* The two-way interaction of joint cue x individual cue, and the main effects of joint cue, individual cue and action-relation were significant. All other interactions were not significant. Greenhouse-Geisser corrections were applied.

Table B.3. ANOVA Table Experiment 1, Error Rates

| Effect | *df1* | *df2* | *F* | *p* | *Partial η²* |
| --- | --- | --- | --- | --- | --- |
| Joint Cue | 1.00 | 23.00 | 1.489 | .235 | 0.061 |
| Individual Cue | 1.53 | 35.15 | 7.222 | .005 | 0.239 |
| Action-Relation | 1.00 | 23.00 | 3.040 | .095 | 0.117 |
| Joint Cue × Individual Cue | 1.12 | 25.87 | 3.192 | .082 | 0.122 |
| Joint Cue × Action-Relation | 1.00 | 23.00 | 1.799 | .193 | 0.073 |
| Individual Cue × Action-Relation | 1.31 | 30.03 | 0.064 | .864 | 0.003 |
| Joint Cue × Individual Cue × Action-Relation | 1.62 | 37.18 | 2.673 | .092 | 0.104 |

*Note.* The main effects of individual cue was significant. All other main effects and interactions were not significant. Greenhouse-Geisser corrections were applied.

Table B.4. ANOVA Table Experiment 2, Three-way ANOVA, main analysis IES

| Effect | *df1* | *df2* | *F* | *p* | *Partial η²* |
| --- | --- | --- | --- | --- | --- |
| Joint Cue | 1.00 | 23.00 | 9.152 | .006 | 0.285 |
| Individual Cue | 1.30 | 29.00 | 30.259 | <.001 | 0.568 |
| Block Validity | 1.00 | 23.00 | 1.608 | .022 | 0.065 |
| Joint Cue × Individual Cue | 1.83 | 42.03 | 1.677 | .201 | 0.068 |
| Joint Cue × Block Validity | 1.00 | 23.00 | 0.260 | .615 | 0.011 |
| Individual Cue × Block Validity | 1.24 | 28.60 | 6.211 | .014 | 0.213 |
| Joint Cue × Individual Cue × Block Validity | 1.74 | 39.90 | 3.907 | .030 | 0.145 |

*Note.* The three-way interaction of joint cue, individual cue and block validity was significant, the two-way interaction of individual cue and block validity was significant, and the main effects of joint cue, individual cue and block validity were significant.

Table B.5. ANOVA Table Experiment 2, Three-way ANOVA, RT

| Effect | *df1* | *df2* | *F* | *p* | *Partial η²* |
| --- | --- | --- | --- | --- | --- |
| Joint Cue | 1.00 | 23.00 | 10.616 | .003 | 0.316 |
| Individual Cue | 1.41 | 32.44 | 24.558 | <.001 | 0.516 |
| Block Validity | 1.00 | 23.00 | 1.313 | .264 | 0.054 |
| Joint Cue × Individual Cue | 1.76 | 40.52 | 0.708 | .482 | 0.030 |
| Joint Cue × Block Validity | 1.00 | 23.00 | 0.830 | .372 | 0.035 |
| Individual Cue × Block Validity | 1.37 | 31.47 | 3.513 | .058 | 0.132 |
| Joint Cue × Individual Cue × Block Validity | 1.91 | 43.84 | 4.529 | .018 | 0.165 |

*Note.* The three-way interaction of joint cue, individual cue and block validity was significant, and the main effects of joint cue and individual cue were significant.

Table B.6. ANOVA Table Experiment 2, Three-way ANOVA, Error Rates

| Effect | *df1* | *df2* | *F* | *p* | *Partial η²* |
| --- | --- | --- | --- | --- | --- |
| Joint Cue | 1.00 | 23.00 | 0.791 | .383 | 0.033 |
| Individual Cue | 1.38 | 31.66 | 4.494 | .031 | 0.163 |
| Block Validity | 1.00 | 23.00 | 0.667 | .423 | 0.028 |
| Joint Cue × Individual Cue | 1.56 | 35.78 | 2.651 | .096 | 0.103 |
| Joint Cue × Block Validity | 1.00 | 23.00 | 1.557 | .225 | 0.063 |
| Individual Cue × Block Validity | 1.51 | 34.69 | 2.242 | .133 | 0.089 |
| Joint Cue × Individual Cue × Block Validity | 1.34 | 30.92 | 0.186 | .742 | 0.008 |

*Note.* The main effect of individual cue was significant.

Table B.7. ANOVA Table Experiment 2, Two-way ANOVA, 100% Individual Cue Validity, IES

| Effect | *df1* | *df2* | *F* | *p* | *Partial η²* |
| --- | --- | --- | --- | --- | --- |
| Joint Cue | 1.00 | 23.00 | 6.530 | .018 | 0.221 |
| Individual Cue | 1.26 | 28.97 | 27.179 | <.001 | 0.542 |
| Joint Cue × Individual Cue | 1.91 | 4.905 | 4.905 | .013 | 0.176 |

*Note.* The two-way interaction and the two main effects of joint cue and individual cue were significant.

Table B.8. ANOVA Table Experiment 2, Two-way ANOVA, 100% Individual Cue Validity, RT

| Effect | *df1* | *df2* | *F* | *p* | *Partial η²* |
| --- | --- | --- | --- | --- | --- |
| Joint Cue | 1.00 | 23.00 | 7.545 | .011 | 0.247 |
| Individual Cue | 1.44 | 33.1 | 21.271 | <.001 | 0.480 |
| Joint Cue × Individual Cue | 1.70 | 39.1 | 3.984 | .033 | 0.148 |

*Note.* The two-way interaction and the two main effects of joint cue and individual cue were significant.

Table B9. ANOVA Table Experiment 2, Two-way ANOVA, 100% Individual Cue Validity, Error Rates

| Effect | *df1* | *df2* | *F* | *p* | *Partial η²* |
| --- | --- | --- | --- | --- | --- |
| Joint Cue | 1.00 | 23.00 | 0.097 | .758 | 0.004 |
| Individual Cue | 1.42 | 32.74 | 5.339 | .017 | 0.188 |
| Joint Cue × Individual Cue | 1.85 | 42.66 | 1.860 | .170 | 0.075 |

*Note.* The main effect of individual cue was significant.

Table B.10. ANOVA Table Experiment 2, Two-way ANOVA, 75% Individual Cue Validity, IES

| Effect | *df1* | *df2* | *F* | *p* | *Partial η²* |
| --- | --- | --- | --- | --- | --- |
| Joint Cue | 1.00 | 23.00 | 4.388 | .047 | 0.160 |
| Individual Cue | 1.40 | 32.10 | 15.961 | <.001 | 0.410 |
| Joint Cue × Individual Cue | 1.92 | 44.25 | 0.537 | .582 | 0.023 |

*Note.* The main effects of joint cue and individual cue were significant.

Table B.11. ANOVA Table Experiment 2, Two-way ANOVA, 75% Individual Cue Validity, RT

| Effect | *df1* | *df2* | *F* | *p* | *Partial η²* |
| --- | --- | --- | --- | --- | --- |
| Joint Cue | 1.00 | 23.00 | 4.400 | .047 | 0.161 |
| Individual Cue | 1.27 | 29.31 | 13.579 | <.001 | 0.371 |
| Joint Cue × Individual Cue | 1.93 | 44.31 | 1.011 | .369 | 0.042 |

*Note.* The main effects of joint cue and individual cue was significant.

Table B.12. ANOVA Table Experiment 2, Two-way ANOVA, 75% Individual Cue Validity, Error Rates

| Effect | *df1* | *df2* | *F* | *p* | *Partial η²* |
| --- | --- | --- | --- | --- | --- |
| Joint Cue | 1.00 | 23.00 | 1.855 | .186 | 0.075 |
| Individual Cue | 1.61 | 37.05 | 0.235 | .744 | 0.010 |
| Joint Cue × Individual Cue | 1.48 | 33.96 | 1.000 | .356 | 0.042 |

*Note.* The main effects and interaction were not significant.

Table B.13. ANOVA Table Experiment 3, IES

| Effect | *df1* | *df2* | *F* | *p* | *Partial η²* |
| --- | --- | --- | --- | --- | --- |
| Joint Cue | 1 | 23 | 16.970 | <.001 | 0.425 |
| Individual Cue | 1 | 23 | 24.000 | <.001 | 0.511 |
| Order | 1 | 23 | 9.359 | .006 | 0.289 |
| Joint Cue × Individual Cue | 1 | 23 | 13.166 | .001 | 0.364 |
| Joint Cue × Order | 1 | 23 | 4.898 | .037 | 0.176 |
| Individual Cue × Order | 1 | 23 | 9.990 | .004 | 0.303 |
| Joint Cue × Individual Cue × Order | 1 | 23 | 0.509 | .483 | 0.022 |

*Note*. All two-way interactions and main effects were significant. The three-way interaction of joint cue, individual cue and cue order was not significant.

Table B.14. ANOVA Table Experiment 3, RT

| Effect | *df1* | *df2* | *F* | *p* | *Partial η²* |
| --- | --- | --- | --- | --- | --- |
| Joint Cue | 1 | 23 | 26.570 | <.001 | 0.536 |
| Individual Cue | 1 | 23 | 25.691 | <.001 | 0.528 |
| Order | 1 | 23 | 13.461 | .001 | 0.369 |
| Joint Cue × Individual Cue | 1 | 23 | 8.958 | .006 | 0.280 |
| Joint Cue × Order | 1 | 23 | 9.431 | .005 | 0.291 |
| Individual Cue × Order | 1 | 23 | 12.525 | .002 | 0.353 |
| Joint Cue × Individual Cue × Order | 1 | 23 | 2.602 | .120 | 0.102 |

*Note*. All two-way interactions and main effects were significant. The three-way interaction of joint cue, individual cue and cue order was not significant.

Table B.15. ANOVA Table Experiment 3, Error Rate

| Effect | *df1* | *df2* | *F* | *p* | *Partial η²* |
| --- | --- | --- | --- | --- | --- |
| Joint Cue | 1 | 23 | 0.566 | .459 | 0.024 |
| Individual Cue | 1 | 23 | 5.522 | .028 | 0.194 |
| Order | 1 | 23 | 0.162 | .691 | 0.007 |
| Joint Cue × Individual Cue | 1 | 23 | 0.042 | .839 | 0.002 |
| Joint Cue × Order | 1 | 23 | 0.190 | .667 | 0.008 |
| Individual Cue × Order | 1 | 23 | 0.007 | .935 | 0.000 |
| Joint Cue × Individual Cue × Order | 1 | 23 | 5.446 | .029 | 0.191 |

*Note*. All two-way interactions and main effects were significant. The three-way interaction of joint cue, individual cue and cue order was not significant.

**Supplementary Material C**

Table C.1. Shapiro-Wilk test for Experiment 1

| Joint Cue | Individual Cue | Action-Relation | Statistic*IES* | pIES | Statistic*log(IES)* | *plog(IES)* |
| --- | --- | --- | --- | --- | --- | --- |
| Present | Non-informative | Different | 0.971 | .703 | 0.938 | .150 |
| Present | Non-informative | Same | 0.968 | .608 | 0.971 | .694 |
| Present | Partner | Different | 0.968 | .625 | 0.956 | .359 |
| Present | Partner | Same | 0.976 | .821 | 0.949 | .264 |
| Present | Self | Different | 0.941 | .169 | 0.975 | .799 |
| Present | Self | Same | 0.937 | .139 | 0.965 | .552 |
| Absent | Non-informative | Different | 0.946 | .225 | 0.982 | .932 |
| Absent | Non-informative | Same | 0.941 | 0.169 | 0.963 | .490 |
| Absent | Partner | Different | 0.952 | .303 | 0.73 | .745 |
| Absent | Partner | Same | 0.820 | <.001 | 0.921 | .062 |
| Absent | Self | Different | 0.97 | .019 | 0.952 | .297 |
| Absent | Self | Same | 0.917 | .049 | 0.959 | .415 |

*Note.* p < .05 indicates violation of normality assumption.

Table C.2. Shapiro-Wilk test for Experiment 2

| Joint Cue | Individual Cue | Block Validity | Statistic*IES* | pIES | Statistic*log(IES)* | *plog(IES)* |
| --- | --- | --- | --- | --- | --- | --- |
| Present | Non-informative | 75 % | 0.94 | .159 | 0.961 | .466 |
| Present | Non-informative | 100 % | 0.877 | .007 | 0.977 | .844 |
| Present | Partner | 75 % | 0.94 | .166 | 0.977 | .845 |
| Present | Partner | 100 % | 0.925 | .074 | 0.932 | .108 |
| Present | Self | 75 % | 0.961 | .045 | 0.992 | .999 |
| Present | Self | 100 % | 0.86 | .003 | 0.948 | .247 |
| Absent | Non-informative | 75 % | 0.92 | .059 | 0.975 | .78 |
| Absent | Non-informative | 100 % | 0.945 | 0.207 | 0.98 | .895 |
| Absent | Partner | 75 % | 0.925 | .075 | 0.957 | .376 |
| Absent | Partner | 100 % | 0.943 | .188 | 0.963 | .498 |
| Absent | Self | 75 % | 0.941 | .169 | 0.977 | .83 |
| Absent | Self | 100 % | 0.797 | <.001 | 0.953 | .309 |

*Note.* p < .05 indicates violation of normality assumption.

Table C.3. Shapiro-Wilk test for Experiment 3

| Joint Cue | Individual Cue | Order | Statistic*IES* | pIES | Statistic*log(IES)* | *plog(IES)* |
| --- | --- | --- | --- | --- | --- | --- |
| Present | Non-informative | Joint Cue Second | 0.848 | .002 | 0.932 | .11 |
| Present | Non-informative | Joint Cue First | 0.868 | .004 | 0.943 | .187 |
| Present | Partner | Joint Cue Second | 0.924 | .073 | 0.927 | .084 |
| Present | Partner | Joint Cue First | 0.918 | .054 | 0.922 | .064 |
| Absent | Non-informative | Joint Cue Second | 0.814 | <.001 | 0.921 | .062 |
| Absent | Non-informative | Joint Cue First | 0.842 | .001 | 0.928 | .087 |
| Absent | Partner | Joint Cue Second | 0.882 | .008 | 0.937 | .138 |
| Absent | Partner | Joint Cue First | 0.836 | .001 | 0.923 | .066 |

*Note.* p < .05 indicates violation of normality assumption.

**Supplementary Material D**

Table D.1. Post-hoc comparisons Experiment 1 Individual Cue × Joint Cue Interaction

| Individual Cue | Group 1 | Group 2 | df | t | p | Cohen’s *d* |
| --- | --- | --- | --- | --- | --- | --- |
| Self | Joint Cue Present | Joint Cue Absent | 47 | -0.113 | .911 | 0.02 |
| Non-informative | Joint Cue Present | Joint Cue Absent | 47 | -2.94 | .001 | 0.42 |
| Partner | Joint Cue Present | Joint Cue Absent | 47 | -7.17 | <.001 | 1.04 |

*Note.* Post-hoc comparisons for the individual cue × joint cue interaction. Post-hoc analysis was conducted using paired t-test (Bonferroni corrected) for the effect of joint cue (present vs. absent) within each level of the individual cue (self, non-informative, and partner).

Table D.2. Post-hoc comparisons Experiment 1 Individual Cue Effect

| Group 1 | Group 2 | df | t | p | Cohen’s *d* |
| --- | --- | --- | --- | --- | --- |
| Non-informative | Partner | 95 | 9.00 | <.001 | 0.92 |
| Non-informative | Self | 95 | 25.0 | <.001 | 2.55 |
| Partner | Self | 95 | 15.4 | <.001 | 1.57 |

*Note.* Post-hoc comparisons for the individual cue main effect.

Table D.5. Post-hoc comparisons Experiment 2, 100% Individual Cue Block, Individual Cue × Joint Cue Interaction

| Individual Cue | Group 1 | Group 2 | df | t | p | Cohen’s *d* |
| --- | --- | --- | --- | --- | --- | --- |
| Self | Joint Cue Present | Joint Cue Absent | 23 | -0.641 | .528 | 0.13 |
| Non-informative | Joint Cue Present | Joint Cue Absent | 23 | -1.06 | .302 | 0.215 |
| Partner | Joint Cue Present | Joint Cue Absent | 23 | -3.75 | .001 | 0.77 |

*Note.* Post-hoc comparisons for the individual cue × joint cue interaction of the 100% individual cue validity block. Post-hoc analysis was conducted using paired t-test (Bonferroni corrected) for the effect of joint cue (present vs. absent) within each level of the individual cue (self, non-informative, and partner).

Table D.6. Post-hoc comparisons Experiment 2, 100% Individual Cue Block, Individual Cue Effect

| Group 1 | Group 2 | df | t | p | Cohen’s *d* |  |
| --- | --- | --- | --- | --- | --- | --- |
| Non-informative | Partner | 47 | 4.94 | <.001 | 0.71 |  |
| Non-informative | Self | 47 | 8.09 | <.001 | 1.17 |  |
| Partner | Self | 47 | -6.21 | <.001 | 0.9 |  |

*Note.* Post-hoc comparisons for the individual cue main effect of the 100% individual cue validity block.

Table D.7. Post-hoc comparisons Experiment 2, 75% Individual Cue Block, Individual Cue Effect

| Group 1 | Group 2 | df | t | p | Cohen’s *d* |  |
| --- | --- | --- | --- | --- | --- | --- |
| Non-informative | Partner | 47 | 4.23 | <.001 | 0.61 |  |
| Non-informative | Self | 47 | 6.14 | <.001 | 0.89 |  |
| Partner | Self | 47 | -4.19 | <.001 | 0.6 |  |

*Note.* Post-hoc comparisons for the individual cue main effect of the 75% individual cue validity block.

Table D.8. Post-hoc comparisons Experiment 3 Individual Cue × Joint Cue Interaction

| Individual Cue | Group 1 | Group 2 | df | t | p | Cohen’s *d* |  |
| --- | --- | --- | --- | --- | --- | --- | --- |
| Non-informative | Joint Cue Present | Joint Cue Absent | 47 | -3.48 | .001 | 0.5 |  |
| Partner | Joint Cue Present | Joint Cue Absent | 47 | -5.55 | <.001 | 0.8 |  |

*Note.* Post-hoc comparisons for the individual cue × joint cue interaction. Post-hoc analysis was conducted using paired t-test (Bonferroni corrected) for the effect of joint cue (present vs. absent) within each level of the individual cue (non-informative vs. partner).

Table D.9. Post-hoc comparisons Experiment 3 Joint Cue × Order Interaction

| Joint Cue | Group 1 | Group 2 | df | T | p | Cohen’s *d* |  |
| --- | --- | --- | --- | --- | --- | --- | --- |
| Present | Joint Cue Second | Joint Cue First | 47 | -0.47 | .642 | 0.07 |  |
| Absent | Joint Cue Second | Joint Cue First | 47 | -4.02 | <.001 | 0.58 |  |

*Note.* Post-hoc comparisons for the joint cue × order interaction. Post-hoc analysis was conducted using paired t-test (Bonferroni corrected) for the effect of order (joint cue first vs. joint cue second) within each level of the joint cue (joint cue present vs. joint cue absent).

Table D.10. Post-hoc comparisons Experiment 3 Individual Cue × Order Interaction

| Individual Cue | Group 1 | Group 2 | df | t | p | Cohen’s *d* |  |
| --- | --- | --- | --- | --- | --- | --- | --- |
| Non-informative | Joint Cue Second | Joint Cue First | 47 | -0.14 | .889 | 0.02 |  |
| Partner | Joint Cue Second | Joint Cue First | 47 | -4.35 | <.001 | 0.63 |  |

*Note.* Post-hoc comparisons for the individual cue × order interaction. Post-hoc analysis was conducted using paired t-test (Bonferroni corrected) for the effect of order (joint cue first vs. joint cue second) within each level of the individual cue (non-informative vs. partner).

**Supplementary Material E**

**Original Design**

The original model was a three-way repeated-measures ANOVA with the factors Joint Cue Presence (informative vs. non-informative), Individual Cue (three levels), and Joint Cue Type / Action Relation (same vs. different). The effect of interest for power estimation was the three-way interaction between these factors.

**Rationale for Flattening**

A three-way interaction in a repeated-measures ANOVA can be expressed as a difference of differences of differences. This allows the interaction to be re-expressed as a single dependent variable that can be analyzed using a one-way repeated-measures ANOVA. This transformation is mathematically equivalent and preserves the statistical information contained in the original interaction term.

**Computation of Interaction Scores**

*For each participant and each level of Individual Cue, we computed:*

PresenceEffect_different = Informative_different – Non-informative_different (Joint Cue Effect, Action Effect = Different)

PresenceEffect_same = Informative_same – Non-informative_same (Joint Cue Effect, Action Effect = Same)

Interaction = PresenceEffect_different – PresenceEffect_same

These interaction scores quantify how the effect of Joint Cue Presence differs between action-relation conditions. Interaction scores can then be used to run a one-way ANOVA approximation on the effect of individual cue on interaction scores. This ANOVA replicates our three-way interaction.

**R Code Used**

**Original Three-Way ANOVA:**

aov_ez(

data = newdata, dv = "log_IES", id = "participant",

within = c("joint_cue_presence", "individualCue", "joint_cue_type")

) %>% nice(es = "pes")

## Anova Table (Type 3 tests)
##
## Response: log_IES
## Effect df MSE F
## 1 joint_cue_presence 1, 23 0.03 21.66 ***
## 2 individualCue 1.39, 31.88 0.08 149.12 ***
## 3 joint_cue_type 1, 23 0.01 18.43 ***
## 4 joint_cue_presence:individualCue 1.60, 36.87 0.02 22.96 ***
## 5 joint_cue_presence:joint_cue_type 1, 23 0.01 0.23
## 6 individualCue:joint_cue_type 1.70, 39.04 0.01 0.41
## 7 joint_cue_presence:individualCue:joint_cue_type 1.96, 45.02

0.01 1.11
## pes p.value
## 1 .485 <.001
## 2 .866 <.001
## 3 .445 <.001
## 4 .500 <.001
## 5 .010 .640
## 6 .018 .632
## 7 .046 .339
## ---
## Signif. codes: 0 '***' 0.001 '**' 0.01 '*' 0.05 '+' 0.1 ' ' 1
##
## Sphericity correction method: GG

**Create interaction scores:**

wide_data <- newdata %>%

group_by(participant, individualCue, joint_cue_presence, joint_cue_type) %>%

summarise(log_IES = mean(log_IES), .groups = "drop") %>%

pivot_wider(names_from = c(joint_cue_presence, joint_cue_type), values_from = log_IES) %>%

mutate(

presence_effect_different = informative_different - `non-informative_different`,

presence_effect_same = informative_same - `non-informative_same`,

interaction = presence_effect_different - presence_effect_same

)

**One-way repeated-measures ANOVA on interaction scores:**

aov_ez(

data = wide_data, dv = "interaction", id = "participant",

within = c("individualCue")

) %>% nice(es = "pes")

## Anova Table (Type 3 tests)
##
## Response: interaction
## Effect df MSE F pes p.value
## 1 individualCue 1.96, 45.02 0.03 1.11 .046 .339
## ---
## Signif. codes: 0 '***' 0.001 '**' 0.01 '*' 0.05 '+' 0.1 ' ' 1
##
## Sphericity correction method: GG

The one-way ANOVA on interaction scores produced identical F values, degrees of freedom, and partial eta-squared values as the three-way interaction in the original ANOVA.

**Power Analysis**

Because G*Power does not support three-way repeated-measures ANOVA directly, the equivalent one-way repeated-measures ANOVA on interaction scores was used to conduct the power analysis.

**G*Power Output – Sample Size**

**
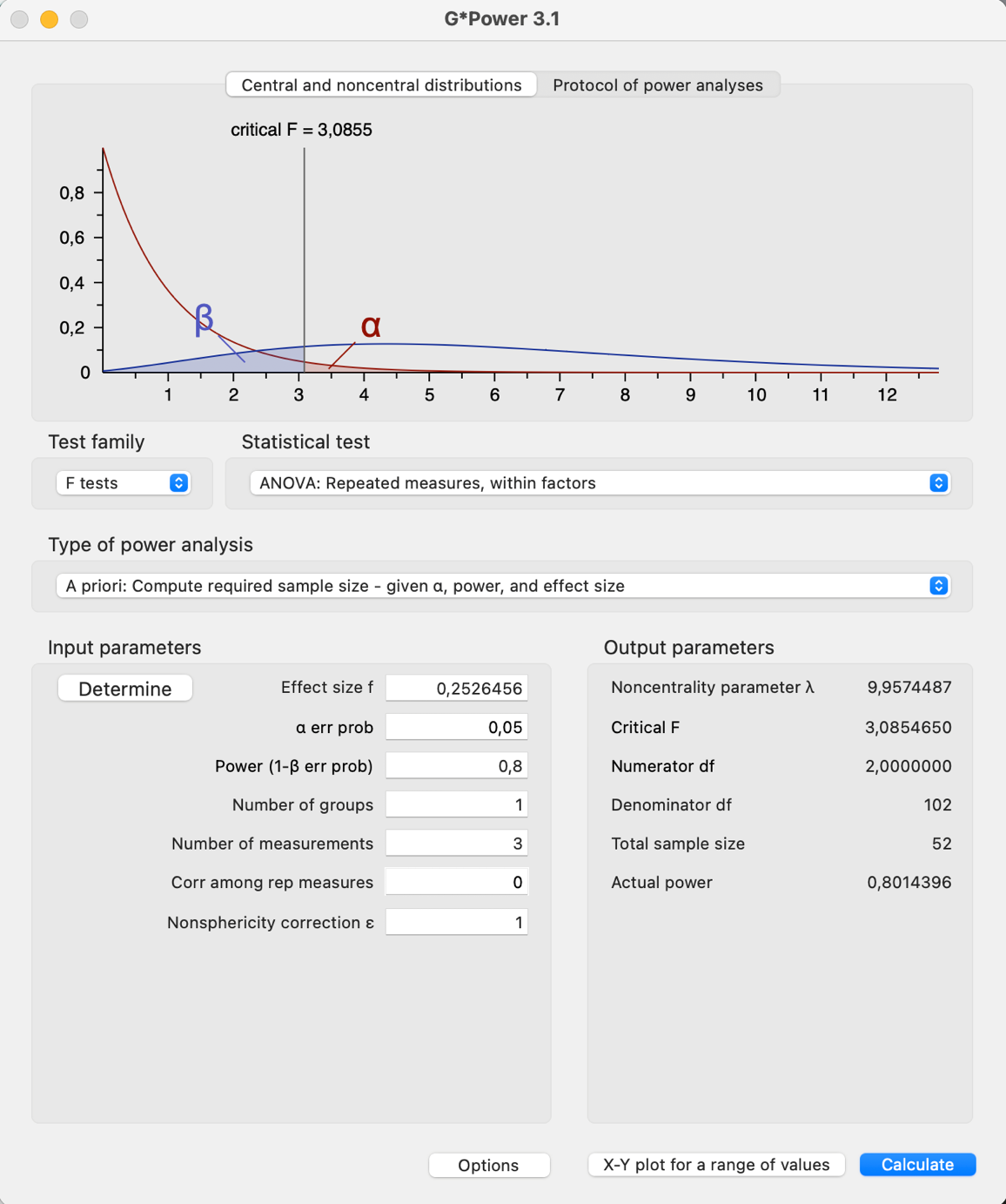
**

**G*Power Output – Post-hoc Sensitivity Analysis**

**
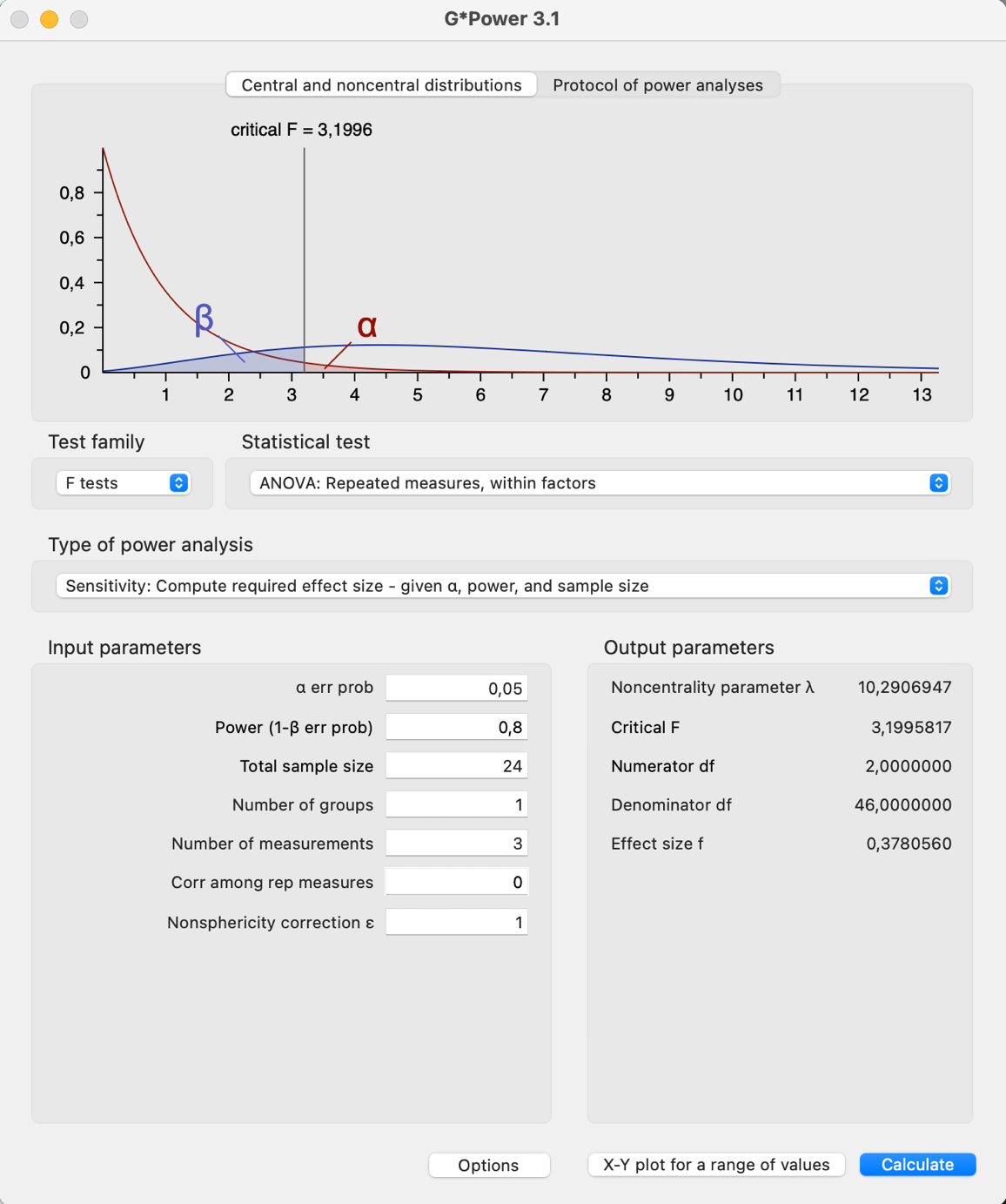
**
